# Comparative Reporting Patterns of Rabeprazole and Other Proton Pump Inhibitors in EudraVigilance: A System Organ Class-Level Disproportionality Analysis

**DOI:** 10.3390/jox16060200

**Published:** 2026-10-19

**Authors:** Radu Dumitru Moleriu, Gabriela Ramona Cioacă, Călin Muntean, Adrian-Cosmin Ilie, Daliborca Cristina Vlad, Teodora Piroș, Ruxandra-Cristina Marin

**Affiliations:** 1 Department III Functional Science, Discipline of Medical Informatics and Biostatistics, “Victor Babes” University of Medicine and Pharmacy, 300041 Timisoara, Romania; radu.moleriu@umft.ro (R.D.M.); teodora.piros@student.umft.ro (T.P.); 2 Doctoral School, “Victor Babes” University of Medicine and Pharmacy, 300041 Timisoara, Romania; 3 Department of Immunology, Allergology and Biology, Department III—Functional Sciences, “Victor Babeș” University of Medicine and Pharmacy, 300041 Timisoara, Romania; cioaca.ramona@umft.ro; 4 Department III Functional Science, Discipline of Public Health and Health Management, “Victor Babeș” University of Medicine and Pharmacy, 300041 Timisoara, Romania; 5 Department IV, Biochemistry and Pharmacology, Discipline of Pharmacology, “Victor Babeș” University of Medicine and Pharmacy, 300041 Timisoara, Romania; vlad.daliborca@umft.ro; 6 Discipline of Pharmacology, Clinical Pharmacology and Pharmacotherapy, “Carol Davila” University of Medicine and Pharmacy, 050474 Bucharest, Romania; ruxandra.marin@umfcd.ro; 7 Doctoral School of Biological and Biomedical Sciences, University of Oradea, 410087 Oradea, Romania

**Keywords:** rabeprazole, proton pump inhibitors, EudraVigilance, disproportionality analysis, reporting odds ratio, System Organ Class, sex-stratified analysis, pharmacovigilance

## Abstract

Background/Objectives: Rabeprazole differs from other proton pump inhibitors (PPIs) in its activation and lower dependence on CYP2C19. We compared its System Organ Class (SOC)-level reporting profile with those of esomeprazole, pantoprazole, omeprazole and lansoprazole in EudraVigilance individual case safety reports (ICSRs). Methods: Aggregated ICSR counts were retrieved from adrreports.eu (data lock point 4 October 2026). Rabeprazole was compared head-to-head with each comparator using reporting odds ratios (RORs) with 95% confidence intervals across 27 MedDRA SOCs, with Benjamini–Hochberg false discovery rate control over all 108 tests. Bonferroni correction and serious-only analyses were sensitivity analyses; female-only and male-only analyses were exploratory. Results: Of 232,045 ICSRs, 7960 involved rabeprazole. Hepatobiliary disorders were the only SOC reported more often with rabeprazole than with all four comparators (ROR 1.29–1.96); the excess met the Bonferroni threshold and persisted among serious reports (ROR 1.19–1.97) and in both sexes. Skin and subcutaneous tissue disorders were reported more often than with three comparators and, among serious reports, than with all four (ROR 1.14–1.83). Eight SOCs showed consistently lower reporting odds, alongside fewer SOCs per rabeprazole ICSR and a different reporter mix. Conclusions: Rabeprazole showed a consistent hepatobiliary reporting signal within the PPI class, in line with earlier FAERS findings. This is a reporting signal, not a clinical signal: without exposure data, the results describe relative reporting, not incidence, comparative risk or causality, and lower reporting odds do not imply lower clinical risk.

## 1. Introduction

Proton pump inhibitors (PPIs) are among the most frequently prescribed and most heavily consumed pharmaceutical agents globally, with established indications spanning gastroesophageal reflux disease, peptic ulcer disease, *Helicobacter pylori* eradication regimens, prevention of nonsteroidal anti-inflammatory drug-associated gastropathy, and pathological hypersecretory conditions [1,2]. By irreversibly inhibiting the gastric H+/K+-ATPase of the parietal cell, all five agents included in the present analysis (esomeprazole, pantoprazole, omeprazole, lansoprazole and rabeprazole) achieve profound and sustained acid suppression [2,3]. The class shares this pharmacodynamic endpoint, yet the individual molecules differ in activation kinetics, potency, and, particularly, metabolic handling. The favorable short-term tolerability of these drugs, combined with broad over-the-counter availability in many jurisdictions, has contributed to sustained and frequently long-term use [3,4].

Rabeprazole has several pharmacological characteristics that distinguish it within the class. Its relatively high pKa favors rapid conversion to the active sulfenamide across the clinically relevant pH range, while a substantial fraction of its clearance occurs through non-enzymatic reduction to a thioether metabolite rather than through cytochrome P450-mediated oxidation [2]. Its dependence on CYP2C19 is consequently lower than that of several other PPIs, reducing the pharmacokinetic variability between extensive and poor metabolizers that is particularly evident with omeprazole [2,3]. The five agents reached clinical use over roughly a decade. Omeprazole was the first PPI and was approved in the United States in 1989 [1,2,5]. Lansoprazole was the second agent of the class [6], with U.S. approval in 1995 [5]. Pantoprazole-containing medicines have been available in the European Union since 1994 [7], and pantoprazole was approved in the United States in 2000 [5]. Rabeprazole was launched first in Japan in 1997, in Europe in 1998 and in the United States in 1999 [5,8]. Esomeprazole, the S-enantiomer of omeprazole, has been authorised in EU countries since 2000 and in the United States since 2001 [5,9]. Rabeprazole is therefore one of the later PPIs and has a smaller representation in spontaneous-reporting data. Despite these pharmacological differences, it remains unclear whether rabeprazole also has a distinct post-marketing reporting profile when evaluated across the full spectrum of adverse-reaction categories.

Despite the generally favorable safety profile of the class, chronic and high-volume PPI use has prompted continued pharmacovigilance attention to a range of potential long-term adverse outcomes. Observational evidence has associated prolonged exposure with hypomagnesemia and other electrolyte disturbances, vitamin B12 deficiency, *Clostridioides difficile*-associated diarrhea and other enteric infections, bone fractures, acute interstitial nephritis, acute kidney injury, and chronic kidney disease, among other outcomes [4,10,11,12,13,14,15,16,17]. Because many of these associations derive from observational designs susceptible to residual confounding and confounding by indication, their magnitude and causal relevance remain debated [10,11]. Spontaneous-reporting systems provide a complementary approach by allowing large-scale assessment of post-marketing reporting patterns and identification of drug–event combinations that may warrant more detailed investigation. Such analyses are primarily hypothesis-generating and complement rather than replace clinical and epidemiological safety studies [18,19,20,21,22].

EudraVigilance, the European Medicines Agency’s centralized system for collecting, managing, and analyzing reports of suspected adverse reactions to medicinal products authorized or studied in the European Economic Area (EEA), constitutes one of the major pharmacovigilance repositories for post-marketing drug safety [23,24]. Its publicly accessible aggregated outputs (adrreports.eu) enable comparative analyses of reporting patterns across structurally and pharmacologically related drugs. Recent EudraVigilance research has also demonstrated the utility of SOC-level pairwise disproportionality analyses for comparing post-marketing reporting profiles among pharmacologically related agents, with sensitivity analyses used to assess the consistency of identified associations [25]. Disproportionality methods, principally the reporting odds ratio (ROR), proportional reporting ratio (PRR), and Bayesian information component (IC), are established quantitative tools for screening spontaneous-reporting databases; they assess whether a particular drug–event combination is reported disproportionately relative to a defined comparator [18,19,20,21]. Importantly, these measures quantify relative reporting rather than event incidence and are sensitive to the composition of both the drug-specific and comparator report pools [22].

Recent real-world-evidence analyses of the U.S. Food and Drug Administration Adverse Event Reporting System (FAERS), the principal U.S. counterpart of EudraVigilance, have extended conventional signal-detection approaches through machine-learning models for signal prediction and prioritization [26,27,28,29]. These developments may complement established pharmacovigilance methods, although conventional disproportionality analysis remains central to the detection and characterization of reporting imbalances in spontaneous-reporting databases. Within-class comparisons, in which agents sharing a therapeutic mechanism serve as one another’s reference, provide a more pharmacologically homogeneous comparator set than an unrestricted database comparator and may reduce some indication-related heterogeneity [21,22]. They do not, however, eliminate differences in prescribing context, cumulative exposure, geography, calendar time, patient characteristics, or reporting behavior between agents and should not be equated with an exposure-adjusted active-comparator epidemiological design.

Previous pharmacovigilance studies of PPIs have mostly examined one outcome at a time, such as hepatotoxicity, acute kidney injury and tubulointerstitial nephritis, neoplasms, hypomagnesaemia or severe cutaneous reactions, and have usually treated the PPIs as a class or compared them with all other drugs in the database [30,31,32,33,34]. EudraVigilance analyses have so far addressed specific populations, for example, paediatric use [35]. To our knowledge, no study has compared rabeprazole head-to-head with each of the other four widely used PPIs across all 27 MedDRA System Organ Classes (SOCs) in EudraVigilance, with multiplicity control over the whole family of comparisons, a serious-only sensitivity analysis and a sex-stratified analysis. A within-class, SOC-wide design shows whether a reporting difference depends on one comparator or recurs against all of them, and it indicates which SOCs deserve Preferred Term (PT)-level work. It complements, and does not replace, PT-level signal detection.

Against this background, the present study places rabeprazole at the centre of a within-class, SOC-oriented comparison of the PPI class in EudraVigilance. We aimed to describe the demographic characteristics, geographic origin, reporter type, seriousness, SOC distribution and reporting complexity of individual case safety reports (ICSRs) for rabeprazole and four comparator PPIs; to quantify rabeprazole-specific differences against each comparator across all 27 SOCs, with false discovery rate (FDR) control over the complete set of comparisons; to identify associations that were consistent in direction and statistical support across all four comparators and preserved among serious reports; and to explore whether these patterns differed between female and male ICSRs. All analyses were descriptive and hypothesis-generating. Disproportionality was read as relative reporting and not as incidence, comparative risk or causality.

## 2. Materials and Methods

### 2.1. Data Source and Extraction

EudraVigilance is the European Medicines Agency system for managing and analyzing reports of suspected adverse reactions to medicines authorized or studied in clinical trials in the European Economic Area. Aggregated and anonymized data are publicly available through the European database of suspected adverse drug reaction reports (adrreports.eu) in accordance with the EudraVigilance access policy [23,24].

Aggregated individual case safety report (ICSR) counts were retrieved for five proton pump inhibitors (PPIs): esomeprazole, pantoprazole, omeprazole, lansoprazole, and rabeprazole. Each agent was searched by active substance in the public interface (Search—Suspected adverse drug reaction reports for Substances), and the corresponding single-substance entry was selected. Fixed-dose combinations, such as esomeprazole with naproxen or acetylsalicylic acid, are listed separately in the interface and were not included. Counts therefore refer to ICSRs in which the selected substance was reported as a suspected or interacting medicinal product [23,24]. For each PPI, substance-level totals were retrieved for all reports and according to age group, sex, geographic origin, reporter group, and seriousness. Cross-tabulations of the 27 Reaction Groups, corresponding to the MedDRA System Organ Classes (SOCs), were also extracted by sex, seriousness, reporter group, geographic origin, and age group from the Number of Individual Cases and Number of Individual Cases by Reaction Groups views in table format. SOC classification followed the MedDRA coding implemented in EudraVigilance. SOC classification followed the MedDRA terminology implemented in EudraVigilance at the data lock point (MedDRA version 29.0).

All five substances were extracted on 7 October 2026 from the same EudraVigilance release, with a data lock point of 4 October 2026. The earliest ICSR available for each substance dated from the first quarter of 2003: 24 January 2003 for omeprazole, 21 February 2003 for lansoprazole, 13 March 2003 for rabeprazole, 14 March 2003 for esomeprazole, and 27 March 2003 for pantoprazole. As EudraVigilance was launched in December 2001 [36], these dates reflect the reporting window represented in the database rather than the market-introduction dates of the individual PPIs. Despite differences in the timing of their clinical introduction, the observable EudraVigilance reporting period was therefore broadly comparable across the five substances.

Geographic origin was classified according to the country in which the suspected reaction occurred (“Occurrence Country” in EudraVigilance). Cases were classified as EEA when the occurrence country was one of the 27 European Union Member States, Iceland, Liechtenstein, or Norway; all other occurrence countries were classified as non-EEA. The public aggregated interface does not provide a country-level breakdown within the non-EEA category.

Duplicate detection and management are performed within EudraVigilance before reports are presented in the aggregated public interface [23,24]. Because the present analysis relied exclusively on publicly available aggregated counts, individual case identifiers were not accessible, and independent deduplication was therefore not possible. For the same reason, reports listing more than one PPI or specific concomitant medicines could not be identified at the individual-case level. Such reports may contribute to more than one substance-specific aggregate, potentially reducing between-drug contrast and introducing dependence between comparison groups; the magnitude and direction of this effect cannot be quantified from aggregated data.

Only aggregated, fully anonymised data were used. No individual-level or patient-identifiable information was accessed, and restricted levels of the EudraVigilance data-analysis environment were not used. Extracted tables were exported as text and checked computationally for internal consistency. Within each cross-tabulation, category counts for every SOC were required to sum to the corresponding SOC total; SOC totals were verified across the five cross-tabulations for each substance; and substance-level totals were reconciled across the extracted views. The final dataset comprised 232,045 ICSRs.

### 2.2. Descriptive Analysis

For each PPI, descriptive proportions were calculated using the substance-specific number of ICSRs as the denominator. Because the proportion of reports with unspecified seriousness differed across PPIs and was highest for rabeprazole, the proportion of serious reports was additionally calculated among ICSRs with known seriousness classification, as serious/(serious + non-serious).

Because a single ICSR may include reactions assigned to more than one SOC, the total number of SOC mentions may exceed the number of ICSRs. Reporting complexity was therefore expressed as the mean number of SOCs reported per ICSR, calculated as the total number of ICSR–SOC mentions divided by the agent-specific ICSR total.

### 2.3. Disproportionality Analysis

Disproportionality was assessed with the reporting odds ratio (ROR) in direct, head-to-head comparisons between two PPIs. The reference group in each comparison was the comparator PPI’s ICSRs; the remaining medicinal products in EudraVigilance were not used as a reference. For a given SOC, the 2 × 2 table was built as follows: *a*, rabeprazole ICSRs reporting at least one reaction in the SOC; *b*, all other rabeprazole ICSRs (total rabeprazole ICSRs minus *a*); *c*, comparator ICSRs reporting the SOC; and *d*, all other comparator ICSRs. The ROR was calculated as (*a*/*b*)/(*c*/*d*) = *ad*/*bc*, with the 95% confidence interval (CI) exp[ln(ROR) ± 1.96 × √(1/*a* + 1/*b* + 1/*c* + 1/*d*)] and a two-sided Wald *p*-value [18,19,21]. The Haldane-Anscombe correction (0.5 added to each cell) was prespecified for tables with a zero cell and was not needed in the all-case analysis.

The same 2 × 2 approach was used to compare seriousness, geographic origin, and reporter type between PPIs. These estimates are reported as odds ratios (ORs), whereas the term ROR is reserved for SOC-level suspected adverse reaction analyses.

Denominators were based on the substance-specific number of ICSRs, either overall or within the relevant stratum, rather than on totals derived from the SOC tables. Because an individual ICSR may contain reactions assigned to more than one SOC, the sum of SOC-specific counts may exceed the total number of ICSRs. Accordingly, the serious-only analyses used the number of serious ICSRs for each substance as the denominator, whereas the sex-stratified analyses used the corresponding numbers of female or male ICSRs. Throughout the manuscript, ROR-based findings are described as disproportionality, reporting excess, or lower reporting odds; they are not interpreted as measures of clinical risk because exposure denominators are unavailable.

Rabeprazole was selected as the index PPI because of its distinctive metabolic profile, characterized by predominantly non-enzymatic clearance and lower dependence on CYP2C19 than other PPIs [2,37]; its lower reporting volume and more limited pharmacovigilance characterization; and previous FAERS findings suggesting a distinct hepatic reporting pattern [30].

Two complementary comparison strategies were applied. First, each PPI was compared with the remaining four PPIs combined across all 27 SOCs to provide a class-level overview of relative reporting patterns. Second, the primary pairwise analysis compared rabeprazole separately with esomeprazole, pantoprazole, omeprazole, and lansoprazole across all 27 SOCs, yielding 108 comparisons.

### 2.4. Multiplicity and Sensitivity Analyses

The primary family comprised 27 SOCs × 4 comparators = 108 pairwise tests. The Benjamini–Hochberg false discovery rate (FDR) procedure was applied across all 108 tests at once [38], and FDR-adjusted *p*-values (q-values) were calculated for each comparison. A Bonferroni threshold based on the same 108 tests (two-sided *p* < 0.05/108 = 4.63 × 10^−4^) was used as a more stringent sensitivity criterion. In the primary all-case analysis, statistical significance was defined as q < 0.05. The serious-only and sex-stratified analyses were treated as separate families of 108 tests and were adjusted independently for multiple testing.

Because individual ICSRs may contain reactions assigned to more than one SOC, the resulting SOC-level tests are not independent. The Benjamini–Hochberg procedure controls the FDR under independence and under certain forms of positive dependence; however, the dependence structure among SOC indicators could not be quantified from the aggregated data and was considered when interpreting the findings.

A study-defined consistency criterion was used to identify associations that were reproducible across comparators. An SOC-level association met this criterion when it was FDR-significant (q < 0.05) and showed the same direction of effect in all four primary pairwise comparisons, with the direction also preserved in the serious-only analysis and the nominal 95% CI excluding 1 in all four comparisons. The use of unadjusted confidence intervals in the serious-only analysis was restricted to this consistency criterion. This criterion was intended to prioritize internally consistent reporting patterns rather than to constitute a validated signal-confirmation framework.

The serious-only sensitivity analysis restricted both the numerator and denominator of each comparison to ICSRs classified as serious.

Associations based on fewer than 50 rabeprazole ICSRs were considered exploratory irrespective of statistical significance. Contextual or procedural MedDRA SOCs, including Social circumstances and Surgical and medical procedures, were distinguished from clinically oriented reaction SOCs when findings were interpreted.

### 2.5. Secondary and Exploratory Analyses

The pairwise analysis was repeated separately for female and male ICSRs across the 27 SOCs and four comparators. SOC-specific numerators were obtained from the SOC × Sex cross-tabulations, and denominators were the corresponding numbers of female or male ICSRs for each substance. Reports with unspecified sex were described but excluded from the stratified analysis. Differences between female and male RORs were assessed using the ratio of RORs (female ROR/male ROR), with 95% CIs and a z-test based on the difference between the corresponding log RORs [39]. Benjamini–Hochberg adjustment was applied across the 108 sex-heterogeneity tests.

Age group, reporter group, and geographic origin were examined descriptively as potential sources of reporting heterogeneity, both at substance level and within SOCs. To further assess the influence of reporter composition, the pairwise analysis was repeated among ICSRs submitted by healthcare professionals, using SOC × Reporter Group counts and the number of HCP-reported ICSRs for each substance as denominators. This post hoc analysis was exploratory and was not incorporated into the study-defined consistency criterion.

Formal temporal disproportionality analyses were not undertaken because the public interface provides cumulative substance-specific counts but does not allow reconstruction of historical SOC-level datasets by reporting period within the same aggregated-data framework.

The main rabeprazole-related disproportionality findings were compared with independent regulatory and published evidence, including adverse reactions described in product information and findings from previous pharmacovigilance and clinical studies.

### 2.6. Software and Ethical Considerations

Statistical analyses were performed using R Statistical Software (Version 4.5.1; R Core Team Foundation for Statistical Computing, Vienna, Austria, 2025), within the RStudio environment (Version 2024.12.1; Posit Team, Boston, MA, USA, 2025), and JASP (Version 0.98.8; JASP Team University of Amsterdam, Amsterdam, The Netherlands). Figures were generated using R and JASP based directly on the validated source data, without imputation or extrapolation.

The study used exclusively aggregated, anonymized, publicly available pharmacovigilance data and involved no human participants, identifiable personal information, or animals. Institutional review board approval and informed consent were therefore not required.

## 3. Results

### 3.1. Overall Characteristics of the ICSR Dataset

**Figure 1:**
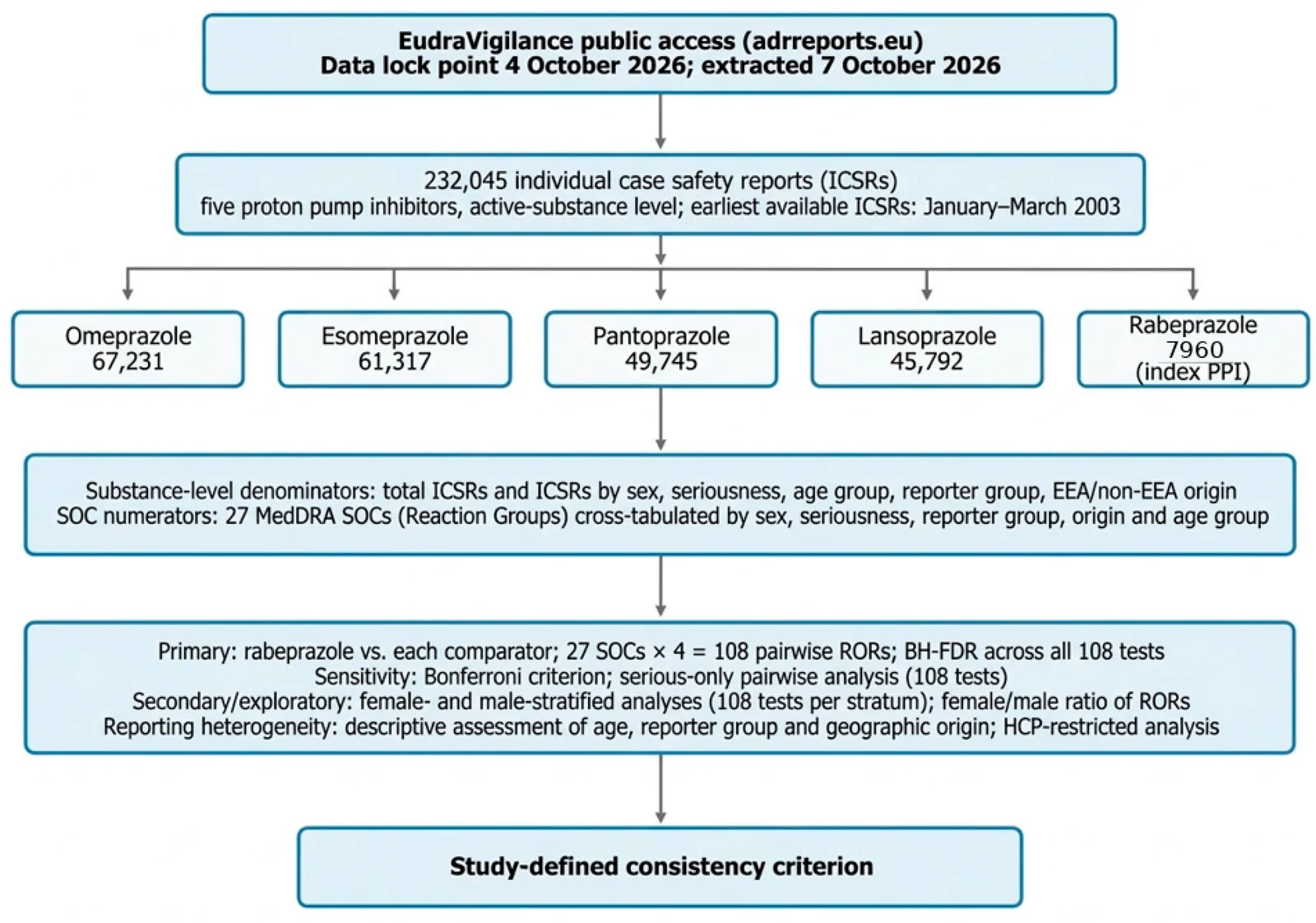
Flow of data retrieval and analytical strategy. Aggregated data were retrieved from the EudraVigilance public access database (adrreports.eu; data lock point: 4 October 2026) for five proton pump inhibitors (PPIs). Substance-level totals provided the denominators, and System Organ Class (SOC) cross-tabulations provided the numerators for the primary, sensitivity, secondary/exploratory, and reporting heterogeneity analyses.

A total of 232,045 ICSRs were identified for the five PPIs (Figure 1). Omeprazole accounted for the largest number of reports (*n* = 67,231; 29.0%), followed by esomeprazole (*n* = 61,317; 26.4%), pantoprazole (*n* = 49,745; 21.4%), and lansoprazole (*n* = 45,792; 19.7%). Rabeprazole had the lowest reporting volume (*n* = 7960; 3.4%).

The overall characteristics of the reports are summarized in Table 1. Adults aged 18–64 years and those aged 65–85 years represented the largest age-defined groups for all five PPIs. Age was unspecified in 33.2–41.5% of reports; among reports with known age, the proportion involving patients aged 65–85 years was highest for rabeprazole (40.2%, compared with 33.2–36.2% for the other PPIs).

Female reports outnumbered male reports for all five agents. Rabeprazole had the lowest proportion of female reports (44.8%) and the highest proportion with unspecified sex (21.4%), followed by lansoprazole (17.5%).

**Figure 2:**
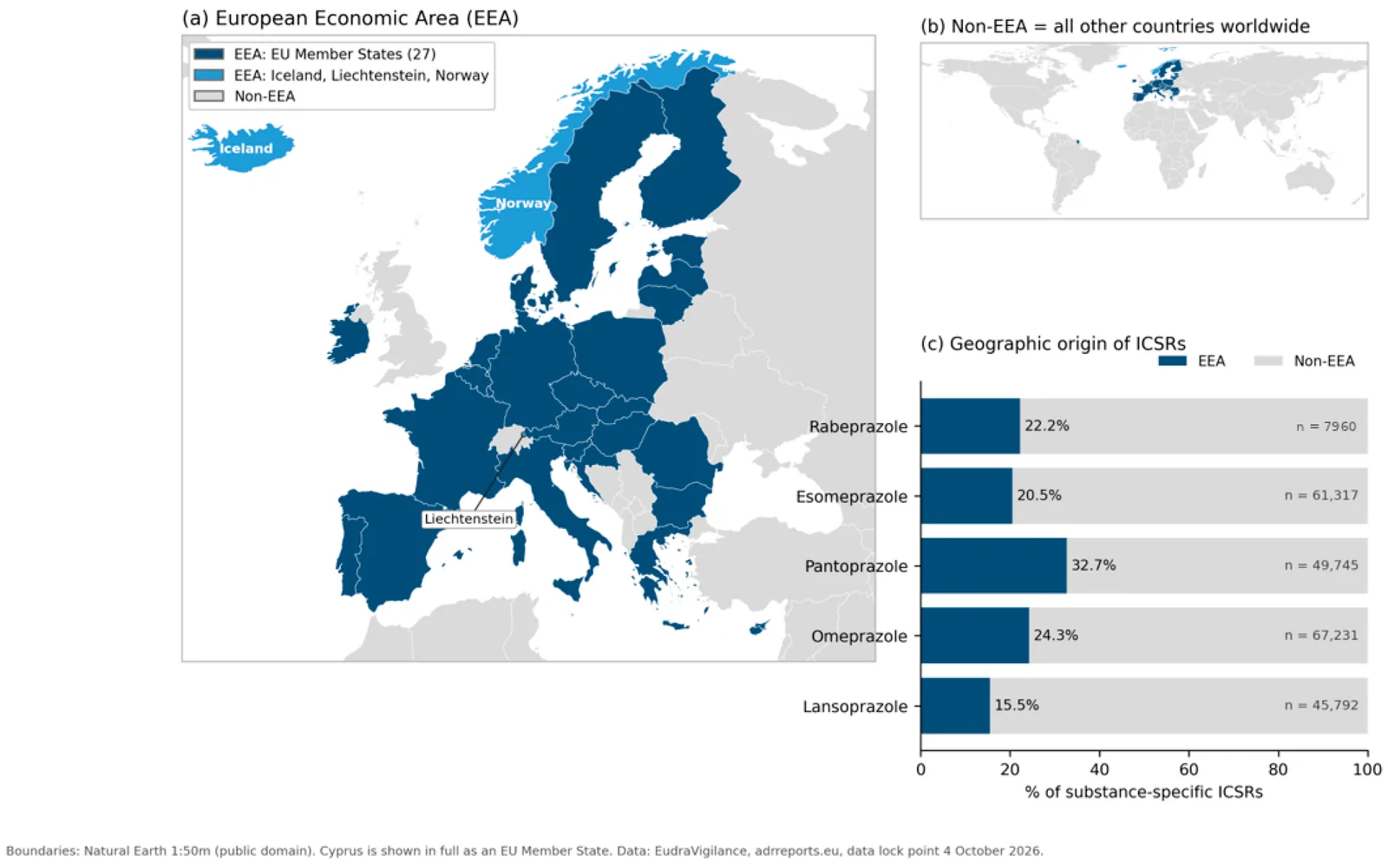
Geographic classification and distribution of EudraVigilance individual case safety reports for the five proton pump inhibitors. (**a**) Countries comprising the European Economic Area (EEA): the 27 European Union Member States, Iceland, Liechtenstein, and Norway. (**b**) All other countries are classified as non-EEA; the map illustrates the geographic definition of this category and does not represent country-specific reporting frequencies. (**c**) Proportions of EEA and non-EEA ICSRs for each PPI.

Geographic origin also differed across the five PPIs (Figure 2). EEA reports accounted for 15.5–32.7% of substance-specific ICSRs, including 22.2% of rabeprazole reports; thus, most reports for every PPI originated outside the EEA. Healthcare professionals submitted 58.9% of rabeprazole ICSRs, the highest proportion among the five agents, compared with 45.7–55.7% for the other PPIs.

Most ICSRs were classified as serious, ranging from 85.8% to 93.1% of substance-specific reports. Unspecified seriousness was more frequent for rabeprazole (3.2%) than for the other PPIs (0.1–0.5%); among reports with a known seriousness classification, 89.7% of rabeprazole ICSRs were serious.

Reporting complexity also varied across agents. Rabeprazole had the lowest mean number of SOCs represented per ICSR (1.72), compared with 1.87–2.26 for the other PPIs (Table 1).

### 3.2. Distribution of Reports by System Organ Class (SOC)

The distribution of ICSRs across the 27 MedDRA System Organ Classes (SOCs) is shown in Table 2. The Renal and urinary disorders SOC was the most frequently represented for all five PPIs, accounting for 43.6% of rabeprazole ICSRs and 46.5–62.6% of comparator reports. Gastrointestinal, general and administration-site, skin and subcutaneous tissue, and nervous-system disorders were also among the most frequently reported SOCs across the five agents.

Rabeprazole had the highest proportion of ICSRs involving Hepatobiliary disorders (5.8% vs. 3.1–4.6% for the comparators). Skin and subcutaneous tissue disorders were also relatively frequent for rabeprazole (13.5%), similar to pantoprazole (13.4%) and higher than for the other three PPIs (8.6–9.6%). For most other SOCs, rabeprazole proportions were similar to or lower than those of the comparator PPIs. Because a single ICSR may contain reactions assigned to more than one SOC, SOC-specific percentages are not mutually exclusive and therefore do not sum to 100%.

### 3.3. Primary Disproportionality Analysis

**Figure 3:**
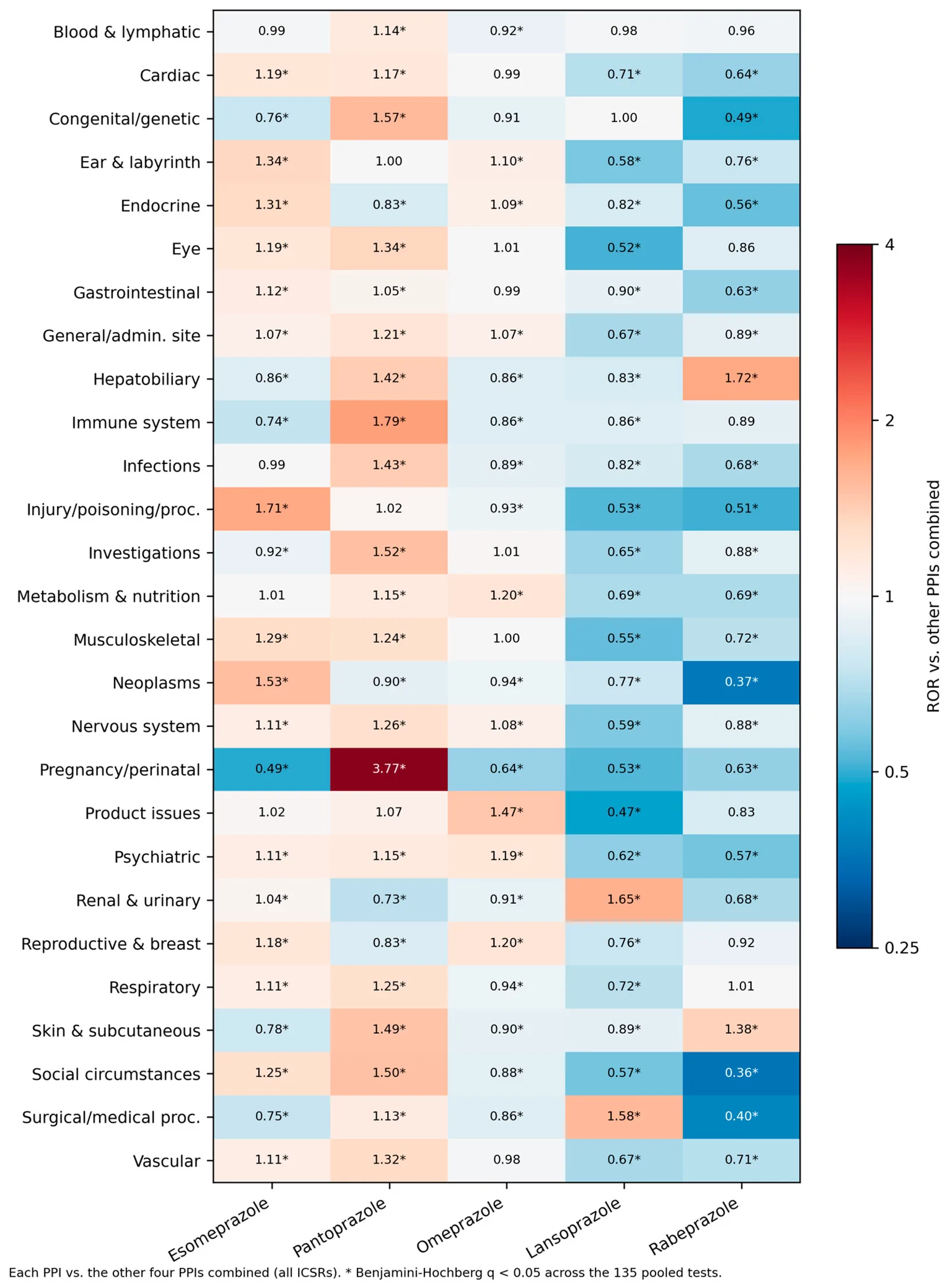
Pooled-comparator analysis of System Organ Class-level reporting patterns across the five proton pump inhibitors. For each PPI, reporting odds ratios (RORs) compare the selected agent with the other four PPIs combined across the 27 MedDRA System Organ Classes (SOCs). Cell values show RORs; colours represent log2(ROR), with values above 1 indicating higher and values below 1 lower relative reporting. Asterisks indicate Benjamini–Hochberg FDR-adjusted q < 0.05 across the 135 pooled comparisons.

The pooled-comparator analysis provided an overview of SOC-level reporting patterns across the five PPIs (Figure 3). The most prominent patterns of higher relative reporting included Renal and urinary disorders for lansoprazole (ROR 1.65, 95% CI 1.62–1.69), Hepatobiliary disorders for rabeprazole (ROR 1.72, 95% CI 1.56–1.89), and Skin and subcutaneous tissue disorders for pantoprazole and rabeprazole (ROR 1.49, 95% CI 1.44–1.53 and ROR 1.38, 95% CI 1.29–1.47, respectively). The complete pooled-comparator profile is shown in Figure 3.

**Figure 4:**
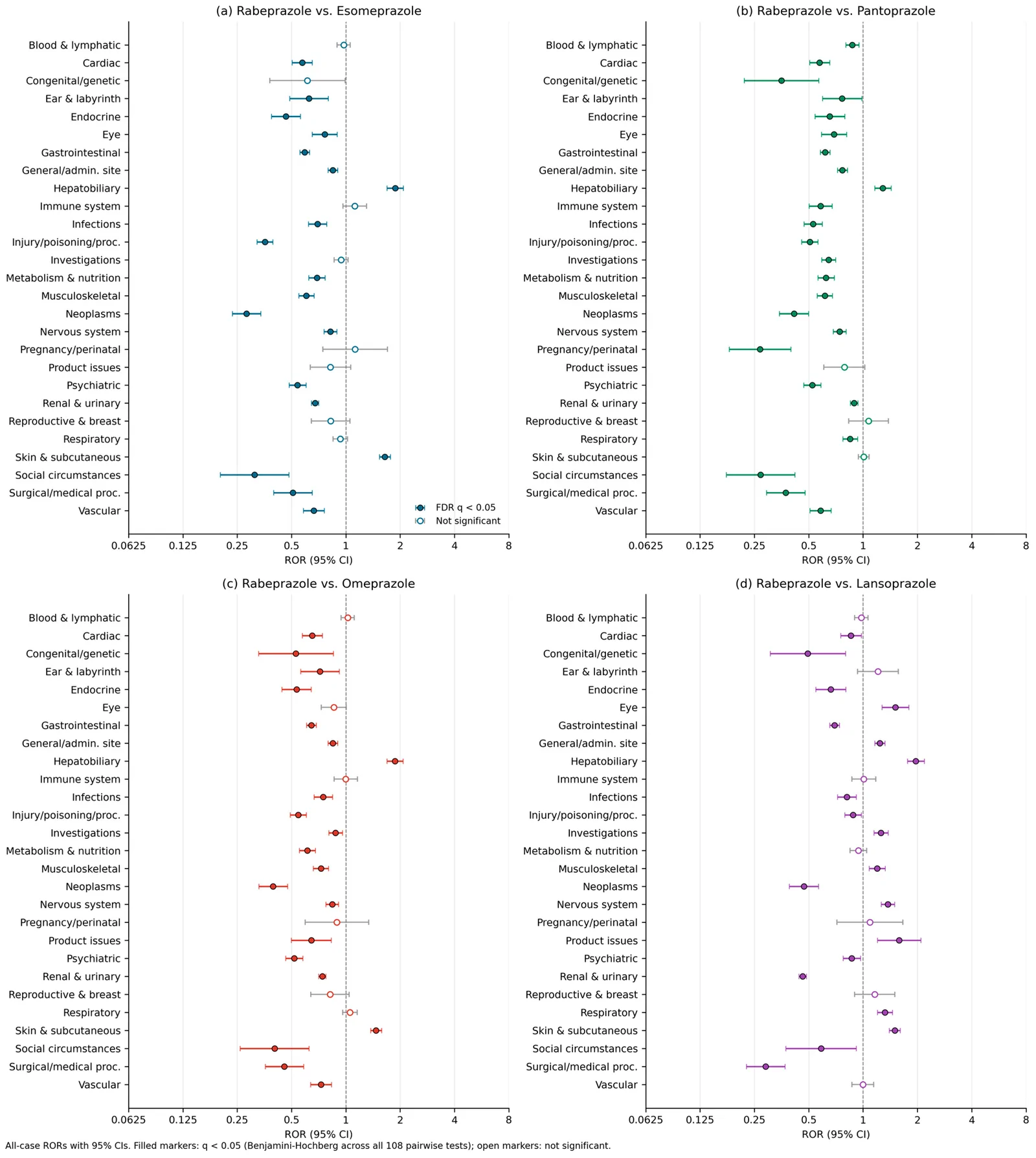
Pairwise System Organ Class-level reporting odds ratios for rabeprazole versus the four comparator proton pump inhibitors. Forest plots show all-case RORs with 95% confidence intervals across the 27 MedDRA System Organ Classes for rabeprazole versus (**a**) esomeprazole, (**b**) pantoprazole, (**c**) omeprazole, and (**d**) lansoprazole. Filled markers indicate FDR-adjusted q < 0.05 after Benjamini–Hochberg correction across the complete family of 108 pairwise tests; open markers indicate non-significant comparisons. The vertical dashed line denotes ROR = 1.

In the primary pairwise analysis, rabeprazole was compared separately with esomeprazole, pantoprazole, omeprazole, and lansoprazole across all 27 SOCs (Figure 4). Complete results for the 108 comparisons are provided in Supplementary Table S1.

Hepatobiliary disorders showed consistently higher reporting odds for rabeprazole than for all four comparators: esomeprazole (ROR 1.88, 95% CI 1.69–2.08), pantoprazole (ROR 1.29, 95% CI 1.16–1.43), omeprazole (ROR 1.87, 95% CI 1.69–2.07), and lansoprazole (ROR 1.96, 95% CI 1.76–2.18). All four comparisons remained significant after FDR adjustment (q < 0.001) and met the Bonferroni criterion. Skin and subcutaneous tissue disorders also showed higher reporting odds relative to esomeprazole (ROR 1.65, 95% CI 1.53–1.77), omeprazole (ROR 1.47, 95% CI 1.37–1.57), and lansoprazole (ROR 1.50, 95% CI 1.39–1.61), whereas no difference was observed relative to pantoprazole (ROR 1.01, 95% CI 0.94–1.08).

**Figure 5:**
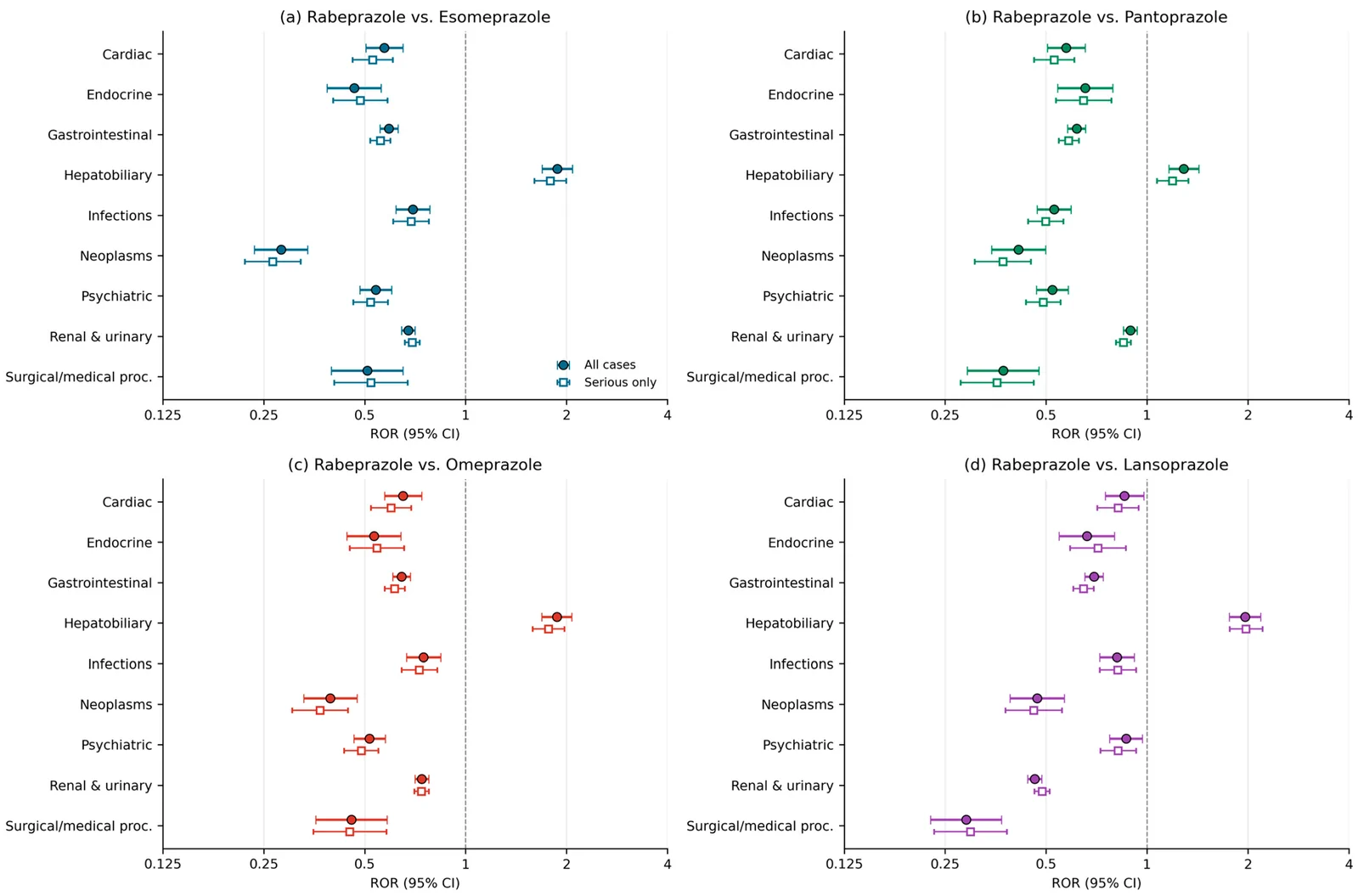
Forest plots of the nine SOC-level associations meeting the study-defined consistency criterion: (**a**) rabeprazole versus esomeprazole; (**b**) rabeprazole versus pantoprazole; (**c**) rabeprazole versus omeprazole; and (**d**) rabeprazole versus lansoprazole. Filled circles show all-case reporting odds ratios (RORs) and open squares serious-only RORs; horizontal lines are 95% confidence intervals. The vertical dashed line indicates ROR = 1.

Nine SOC-level associations met the study-defined consistency criterion (Table 3; Figure 5). Hepatobiliary disorders was the only SOC with consistently higher reporting odds for rabeprazole across all four comparators (ROR range 1.29–1.96). Eight SOCs showed consistently lower reporting odds: Cardiac disorders, Endocrine disorders, Gastrointestinal disorders, Infections and infestations, Neoplasms, Psychiatric disorders, Renal and urinary disorders, and Surgical and medical procedures. Six associations (Endocrine disorders, Gastrointestinal disorders, Hepatobiliary disorders, Neoplasms, Renal and urinary disorders, and Surgical and medical procedures) also met the Bonferroni criterion in all four comparisons.

### 3.4. Robustness and Reporting-Heterogeneity Analyses

The serious-only analysis broadly confirmed the main findings (Table 4; Supplementary Table S2). All nine associations meeting the study-defined consistency criterion retained the same direction of effect. The hepatobiliary reporting excess persisted against all four comparators (ROR 1.19–1.97), while Skin and subcutaneous tissue disorders showed higher reporting odds against all four comparators in the serious-only analysis (ROR 1.14–1.83), including pantoprazole, for which no difference was observed in the all-case analysis.

Reporting composition differed across SOCs (Supplementary Table S3). Renal and urinary disorders were characterized by predominantly non-HCP and non-EEA reporting; among rabeprazole renal reports, only 22.3% were submitted by HCPs and 97.3% originated outside the EEA. In the HCP-restricted analysis, the hepatobiliary excess persisted against all four comparators (ROR 1.26–1.79), while the cutaneous excess remained against three comparators. The lower gastrointestinal reporting odds were substantially attenuated (ROR 0.83–1.04), whereas the lower renal reporting odds persisted and became more pronounced (ROR 0.26–0.49) (Table 4; Supplementary Table S4).

**Figure 6:**
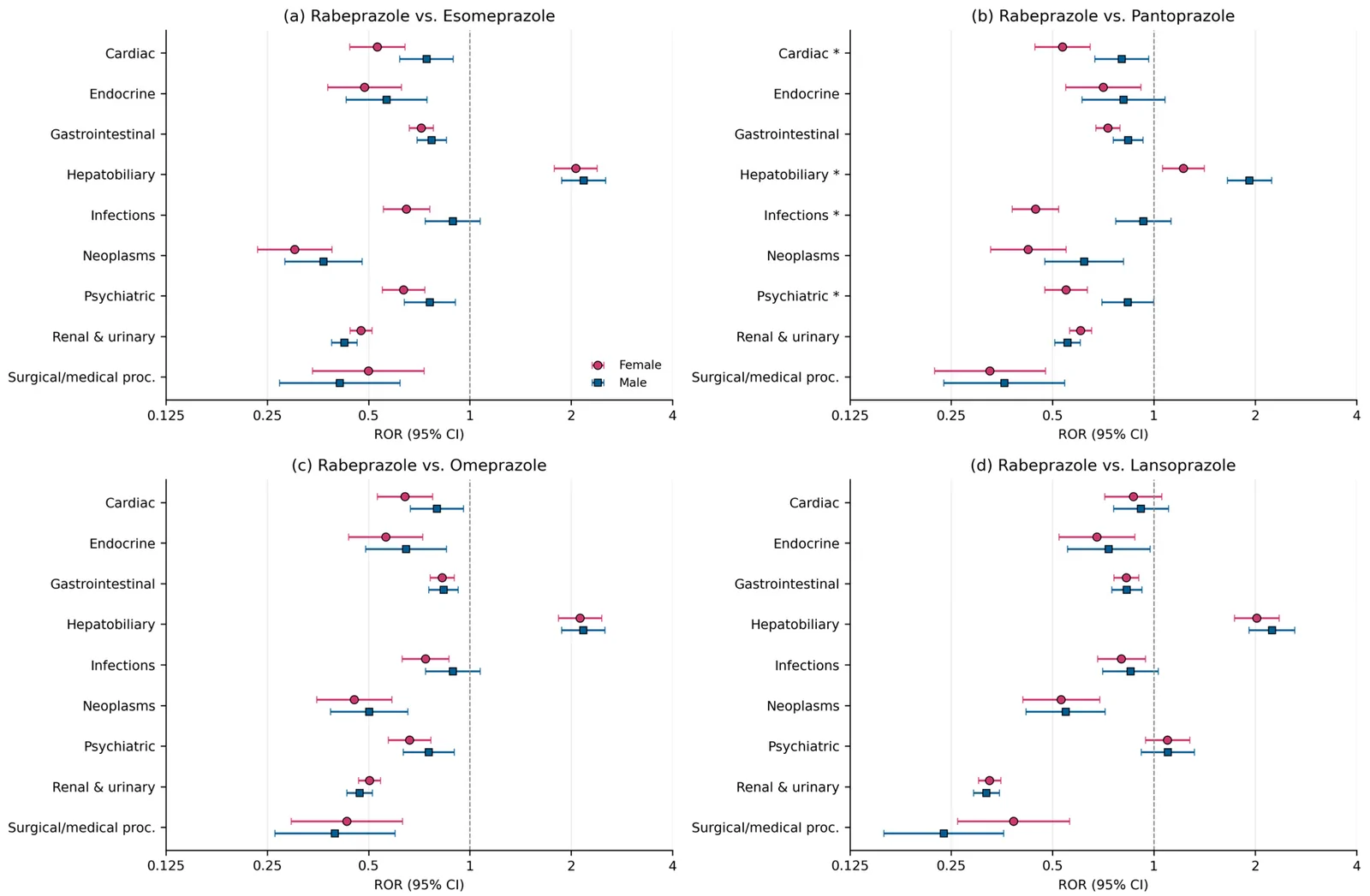
Sex-stratified reporting odds ratios (RORs) with 95% confidence intervals for Hepatobiliary disorders, Skin and subcutaneous tissue disorders and the SOCs meeting the consistency criterion: (**a**) rabeprazole versus esomeprazole; (**b**) versus pantoprazole; (**c**) versus omeprazole; and (**d**) versus lansoprazole. Circles, female ICSRs; squares, male ICSRs. An asterisk marks a significant female/male difference (q < 0.05). The vertical dashed line indicates the null value (ROR = 1), representing equal reporting odds between rabeprazole and the comparator.

Sex-stratified analyses are shown in Figure 6 and reported in full in Supplementary Table S5. The hepatobiliary excess was present in both female and male ICSRs against all four comparators (female RORs 1.22–2.12; male RORs 1.92–2.24). A significant female–male difference was observed only versus pantoprazole (ratio of RORs 0.64, 95% CI 0.52–0.78; q < 0.001). The cutaneous excess was also observed in both sexes, whereas lower reporting of Infections and infestations was confined to female reports.

### 3.5. Cross-Source Contextualization of Key SOC-Level Reporting Patterns

The main EudraVigilance reporting patterns were descriptively compared with corresponding information from rabeprazole product information and previously published pharmacovigilance studies (Table 5). This cross-source comparison was intended to provide context for the observed SOC-level patterns and was not used as an additional statistical test or criterion for signal selection.

The comparison focused on Hepatobiliary disorders and Skin and subcutaneous tissue disorders, which showed higher relative reporting for rabeprazole in the present analysis, and on selected SOCs with consistently lower reporting odds. Because the external sources differed in database structure, reporting period, comparator definition, and level of analysis, their findings were considered descriptive rather than directly comparable estimates.

## 4. Discussion

In this within-class analysis of 232,045 EudraVigilance ICSRs for five PPIs, rabeprazole showed an asymmetric reporting profile: one consistent positive disproportionality for Hepatobiliary disorders; a cutaneous excess that was consistent only among serious reports; and lower relative reporting across eight other SOCs. These findings carry different weight. The hepatobiliary association was present against all four comparators, among serious reports, in both sexes and among HCP reports, whereas the negative disproportionalities are harder to interpret because rabeprazole reports differed from those of the comparators in reporting complexity and reporter composition. The differences in reporting volume across PPIs are compatible with differences in utilization, market history and cumulative exposure, which could not be quantified with the available data. RORs compare relative reporting, not exposed populations; they describe reporting imbalance and not incidence or comparative clinical risk [21,22,41,42].

The hepatobiliary reporting excess is the principal positive finding. Rabeprazole showed higher reporting odds than all four comparators (ROR 1.29–1.96), the associations met the Bonferroni threshold over 108 tests, and the direction was preserved among serious reports (ROR 1.19–1.97). Restricting the analysis to HCP reports did not remove the excess, which argues against a purely reporter-driven artefact. Independent pharmacovigilance evidence points the same way. A FAERS signal-mining study of PPI-associated hepatotoxicity identified cholestatic hepatitis, cholestasis and fulminant hepatitis among the leading hepatic Preferred Terms and reported the highest fulminant-hepatitis ROR for rabeprazole (ROR 40.2, 95% CI 20.0–80.8), an estimate the authors considered possibly inflated by small numbers [30]. Hepatic adverse reactions are also recognized in the product information [4]. Concordance between EudraVigilance and FAERS is not formal replication, because the databases differ in reporting populations, periods, comparators and methods, but it strengthens the case for a closer look at the hepatobiliary reports.

The mechanism underlying this pattern cannot be determined from the present data. Rabeprazole differs pharmacokinetically from several other PPIs because its metabolism includes substantial non-enzymatic reduction and is less dependent on CYP2C19 [2,37]. However, this characteristic does not provide a direct explanation for the observed hepatobiliary disproportionality. Drug-induced liver injury is heterogeneous and may involve direct, metabolic, immune-mediated, mitochondrial, or other idiosyncratic mechanisms, and diagnosis requires consideration of the temporal relationship, competing causes, concomitant treatments, and the clinical pattern of liver injury [43]. Accordingly, the present SOC-level association should be considered a pharmacovigilance finding requiring further characterization rather than evidence of a specific rabeprazole-induced hepatotoxic mechanism. Preferred Term-level analysis followed, where possible, by case-level assessment of latency, dechallenge, rechallenge, alternative causes, and concomitant medication would be the appropriate next step [43].

The cutaneous pattern was less consistent and is a secondary finding. In the all-case analysis, rabeprazole showed higher reporting odds for Skin and subcutaneous tissue disorders relative to esomeprazole, omeprazole and lansoprazole (ROR 1.47–1.65) but not pantoprazole (ROR 1.01, 95% CI 0.94–1.08), so this SOC did not meet the consistency criterion. Among serious reports, the excess was present against all four comparators, including pantoprazole (ROR 1.14, 95% CI 1.05–1.23). PPIs are recognized causes of immediate and delayed hypersensitivity reactions, including severe cutaneous manifestations [4,31,40,44], and subacute cutaneous lupus erythematosus and severe cutaneous adverse reactions have been described with this class [4,31,40]. PT-level analysis is needed to identify which cutaneous reactions drive the serious-only pattern. Pantoprazole itself showed one of the highest pooled cutaneous RORs in the class, which explains why it was the comparator against which the all-case difference disappeared.

The eight consistently lower disproportionalities need a different reading and do not indicate lower clinical risk. Rabeprazole ICSRs contained fewer SOCs per report than those of the comparators (mean 1.72 vs. 1.87–2.26), which can lower SOC-specific reporting odds across several categories at once. Reporter composition matters as well. The lower reporting of Gastrointestinal disorders largely disappeared among HCP reports, whereas the cardiac, infectious, neoplastic and procedural deficits persisted. Disproportionality is also sensitive to comparator choice, stimulated or notoriety-related reporting and the distribution of competing events [18,22,41,42,45]. Lower RORs therefore describe relative reporting and should not be read as a safety advantage of rabeprazole in these domains.

The renal findings show this most clearly. Renal and urinary disorders were the most frequently reported SOC for all five PPIs, from 43.6% of rabeprazole ICSRs to 62.6% of lansoprazole ICSRs, and the negative rabeprazole ROR ranged from 0.89 against pantoprazole to 0.46 against lansoprazole. For every PPI, these reports came overwhelmingly from outside the EEA and from non-healthcare professionals, often without age or sex. A profile of this kind is compatible with stimulated reporting, including reports linked to litigation, which has been shown to concentrate on specific drug–event pairs in spontaneous-reporting systems [46,47]. PPIs have been associated with acute kidney injury and tubulointerstitial nephritis in observational and pharmacovigilance studies [12,16,17,32]. The present data do not suggest lower renal risk with rabeprazole; they show that within-class disproportionality can be driven by the concentration of one reporting stream in the comparator drug. Because renal reports make up such a large share of each comparator’s ICSRs, they also shift the denominators of every other SOC, which is a further reason for caution with the negative RORs.

Two further features limit the clinical reading of the negative associations. Surgical and medical procedures is a procedural MedDRA SOC whose disproportionality mainly reflects report structure. In addition, SOCs with fewer than 50 rabeprazole ICSRs, such as Congenital, familial and genetic disorders and Social circumstances, were treated as exploratory; both met the consistency criterion in the original analysis but not in the updated one, which illustrates how unstable estimates based on small counts can be. Statistical significance does not overcome this, and current recommendations ask for case numbers, clinical relevance, comparator choice and supporting evidence to be weighed alongside the size and precision of disproportionality estimates [41,42].

The demographic and reporter characteristics add context. Among ICSRs with known age, rabeprazole had the highest proportion of patients aged 65–85 years (40.2% vs. 33.2–36.2%). Rabeprazole also had the lowest female proportion (44.8%), the highest proportion of unspecified sex (21.4%) and the most HCP-weighted reporter profile (58.9%). Female predominance, concentration of reports among adults and older people and sparse paediatric reporting have been described across pharmacovigilance databases and PPI datasets [4,11,23,24,45,48,49,50]. These distributions cannot separate exposure from susceptibility or reporting behaviour and are descriptive features of the reporting population, not demographic risk estimates.

The sex-stratified analysis was added because PPIs are metabolized by the cytochrome P450 system and because women report adverse drug reactions more often, partly as a result of sex differences in pharmacokinetics and dosing [49,51,52]. The hepatobiliary excess was present in both female and male ICSRs, and the only significant difference between strata, against pantoprazole, reflected a stronger excess in male reports. The cutaneous excess was somewhat larger in female reports against two comparators. The data therefore do not suggest that the principal findings are confined to one sex. Rabeprazole depends less on CYP2C19 than the other PPIs, so CYP-related sex differences would be expected to affect the comparators more than rabeprazole itself; aggregated reports cannot test this. With 108 tests per stratum and a further 108 heterogeneity tests, isolated sex differences, such as the female-only infectious deficit, should be treated as hypotheses.

Seriousness also needs a denominator-aware reading. Although 86.8% of all rabeprazole ICSRs were serious, unspecified seriousness was more frequent for rabeprazole (3.2%) than for the comparators (0.1–0.5%). Among reports with known seriousness, 89.7% of rabeprazole ICSRs were serious, within the class range of 86.1–93.3%, and the odds of serious reporting were similar to those of the pooled comparator. The hepatobiliary and cutaneous serious-only patterns are therefore SOC-specific and not a consequence of a generally higher share of serious rabeprazole reports.

The analysis has several strengths. All comparisons stayed within one pharmacological class; four separate pairwise comparisons showed whether an association depended on a single comparator; multiplicity was controlled over the whole family of 108 tests, with a stricter Bonferroni sensitivity criterion; serious-only, sex-stratified and HCP-restricted analyses tested the stability of the main findings; and the complete 27-SOC results, with all 2 × 2 counts, are reported in the Supplementary Materials. These elements follow current recommendations on transparent comparator definition, complete reporting and cautious interpretation of disproportionality studies [41,42].

The limitations are those of aggregated spontaneous-reporting data. No exposure denominator was available, so incidence, absolute risk and exposure-adjusted comparisons were not possible [21,22,41,42]. Under-reporting, selective and stimulated reporting, notoriety bias, differential reporting across countries and reporter types, and confounding by indication may affect both positive and negative disproportionalities [18,22,41,42,45]. Duplicates were managed only within EudraVigilance, reports naming more than one PPI could not be identified, and missing clinical information, polypharmacy, indication, dose and treatment duration could not be addressed. Market authorization dates, prescribing volume and geographic uptake differ between the five PPIs and could not be adjusted for.

The SOC level is broad, and an association may be driven by one or a few Preferred Terms; this matters most for Hepatobiliary and Skin and subcutaneous tissue disorders, where PT-level characterization is needed. SOC indicators are not independent, and their dependence structure could not be assessed from aggregate data, so the consistency criterion is a transparent way of prioritizing internally consistent findings rather than a validated confirmation procedure [41,42]. Stratified analyses were limited to seriousness, sex and HCP reports; age and geographic origin were described, whereas formal analysis by reporting year was not feasible using the aggregated public interface. Calendar time therefore remains an unmeasured source of reporting heterogeneity.

Rabeprazole had a much smaller reporting base than the other PPIs, which makes estimates for uncommon SOCs sensitive to small changes in counts, as the shift in the two small SOCs between extractions shows. Finally, the dataset reflects cumulative reporting up to the data lock point of 4 October 2026, and estimates may change as exposure, prescribing, regulatory attention and reporting evolve [41,42].

The cross-source comparison supports prioritization but is not external validation. EudraVigilance and FAERS differ in reporting populations, regulatory settings, periods, duplicate management and comparator distributions, and product information and published reports represent different levels of evidence. Recurrence across sources was clearest for Hepatobiliary disorders, supported by previous FAERS findings and recognized hepatic reactions [4,30]. The cutaneous association received more limited support, because its consistency appeared only among serious reports [4,31,40,44].

Future work should prioritize PT-level disproportionality within the hepatobiliary and serious cutaneous strata, followed where possible by case-level review of clinical phenotype, latency, dechallenge and rechallenge, concomitant medication and alternative causes, which is essential for causality assessment in suspected liver injury [43]. Exposure-adjusted epidemiological studies, repeated extractions, comparison with FAERS and other databases, and complementary measures such as the proportional reporting ratio and the information component would show whether the findings depend on the data source or the statistical measure [22,41,42].

Machine-learning methods may later help with signal prioritization in large spontaneous-reporting datasets [27,29], but they complement rather than replace pharmacological assessment, clinical review and established signal-detection methods. For this dataset, more clinical granularity, through PT-level and case-level analyses, is the more useful next step.

Overall, the findings support a focused rather than global reading of the rabeprazole pharmacovigilance profile. Hepatobiliary disorders were the most consistent positive association, supported by all four comparisons, the serious-only, sex-stratified and HCP-restricted analyses, and independent pharmacovigilance evidence [30]. The serious-case cutaneous excess is a secondary finding that warrants targeted investigation. The negative SOC-level associations remain descriptive, because reporting complexity, reporter composition, comparator-specific reporting streams and the lack of exposure denominators prevent their interpretation as comparative safety advantages. These results identify reporting signals for further characterization, not causal or incidence-based differences in safety among PPIs [21,22,41,42].

## 5. Conclusions

Across 232,045 EudraVigilance ICSRs for five PPIs, rabeprazole showed a distinct within-class reporting profile, with a consistent hepatobiliary reporting signal. The reporting excess was present against all four comparators, met the FDR and Bonferroni criteria over 108 tests, and persisted among serious reports, in both sexes and among HCP reports. Its concordance with previous FAERS findings supports investigation at the Preferred Term and individual-case levels. A secondary cutaneous pattern was consistent only among serious reports. These are reporting signals, not clinical signals.

The lower reporting odds observed for eight other SOCs should not be interpreted as lower clinical risk; they are shaped by reporting complexity, reporter composition, comparator-specific reporting streams and the smaller rabeprazole reporting base. Without exposure denominators, incidence, absolute risk and comparative safety cannot be estimated. The findings describe relative reporting patterns and support PT-level, cross-database and exposure-adjusted studies of rabeprazole safety.

## Figures and Tables

**Table 1:** Characteristics of individual case safety reports (ICSRs) for the five proton pump inhibitors in EudraVigilance (data lock point 4 October 2026).

Characteristic	Rabeprazole	Esomeprazole	Pantoprazole	Omeprazole	Lansoprazole
Total ICSRs, *n*	7960	61,317	49,745	67,231	45,792
Sex					
Female	3564 (44.8)	31,878 (52.0)	26,329 (52.9)	34,113 (50.7)	22,037 (48.1)
Male	2693 (33.8)	21,351 (34.8)	17,924 (36.0)	24,429 (36.3)	15,745 (34.4)
Not specified	1703 (21.4)	8088 (13.2)	5492 (11.0)	8689 (12.9)	8010 (17.5)
Female:male ratio	1.32	1.49	1.47	1.40	1.40
Age group					
0–1 month	6 (0.1)	96 (0.2)	53 (0.1)	121 (0.2)	28 (0.1)
2 months-2 years	7 (0.1)	224 (0.4)	49 (0.1)	349 (0.5)	118 (0.3)
3–11 years	11 (0.1)	196 (0.3)	95 (0.2)	360 (0.5)	135 (0.3)
12–17 years	19 (0.2)	235 (0.4)	199 (0.4)	452 (0.7)	152 (0.3)
18–64 years	2775 (34.9)	24,677 (40.2)	19,642 (39.5)	26,102 (38.8)	15,091 (33.0)
65–85 years	2090 (26.3)	13,502 (22.0)	11,460 (23.0)	15,379 (22.9)	9693 (21.2)
>85 years	294 (3.7)	1713 (2.8)	1751 (3.5)	1895 (2.8)	1587 (3.5)
Not specified	2758 (34.6)	20,674 (33.7)	16,496 (33.2)	22,573 (33.6)	18,988 (41.5)
65–85 years, % of age-known	40.2	33.2	34.5	34.4	36.2
Reporter group					
Healthcare professional	4688 (58.9)	28,046 (45.7)	27,706 (55.7)	34,965 (52.0)	24,060 (52.5)
Non-healthcare professional	3259 (40.9)	33,221 (54.2)	21,486 (43.2)	31,924 (47.5)	21,433 (46.8)
Not specified	13 (0.2)	50 (0.1)	553 (1.1)	342 (0.5)	299 (0.7)
Geographic origin					
EEA	1768 (22.2)	12,564 (20.5)	16,253 (32.7)	16,326 (24.3)	7097 (15.5)
Non-EEA	6192 (77.8)	48,753 (79.5)	33,491 (67.3)	50,905 (75.7)	38,695 (84.5)
Not specified	0	0	1	0	0
Seriousness					
Serious	6909 (86.8)	55,472 (90.5)	42,671 (85.8)	59,704 (88.8)	42,648 (93.1)
Non-serious	795 (10.0)	5810 (9.5)	6914 (13.9)	7212 (10.7)	3061 (6.7)
Not specified	256 (3.2)	35 (0.1)	160 (0.3)	315 (0.5)	83 (0.2)
Serious, % of classified	89.7	90.5	86.1	89.2	93.3
Reporting complexity					
ICSR–SOC pairs, *n*	13,674	134,329	112,248	139,682	85,855
Mean number of SOCs per ICSR	1.72	2.19	2.26	2.08	1.87

Values are *n* (% of the substance-specific total) unless stated otherwise. The serious proportion among classified ICSRs was calculated as serious/(serious + non-serious). Mean SOCs per ICSR = total ICSR-SOC mentions divided by the number of ICSRs. EEA, European Economic Area (27 EU Member States, Iceland, Liechtenstein and Norway); non-EEA includes all other countries worldwide.

**Table 2:** Distribution of individual case safety reports (ICSRs) across the 27 MedDRA System Organ Classes (SOCs) for the five proton pump inhibitors.

System Organ Class	Rabeprazole	Esomeprazole	Pantoprazole	Omeprazole	Lansoprazole
Blood and lymphatic system disorders	679 (8.5)	5361 (8.7)	4806 (9.7)	5614 (8.4)	3984 (8.7)
Cardiac disorders	261 (3.3)	3428 (5.6)	2773 (5.6)	3327 (4.9)	1743 (3.8)
Congenital, familial and genetic disorders	18 (0.2)	226 (0.4)	317 (0.6)	287 (0.4)	209 (0.5)
Ear and labyrinth disorders	69 (0.9)	846 (1.4)	562 (1.1)	809 (1.2)	329 (0.7)
Endocrine disorders	120 (1.5)	1954 (3.2)	1137 (2.3)	1878 (2.8)	1035 (2.3)
Eye disorders	170 (2.1)	1704 (2.8)	1524 (3.1)	1669 (2.5)	652 (1.4)
Gastrointestinal disorders	1349 (16.9)	15,743 (25.7)	12,369 (24.9)	16,181 (24.1)	10,392 (22.7)
General disorders and administration site conditions	1354 (17.0)	11,950 (19.5)	10,476 (21.1)	13,096 (19.5)	6505 (14.2)
Hepatobiliary disorders	465 (5.8)	1962 (3.2)	2286 (4.6)	2158 (3.2)	1404 (3.1)
Immune system disorders	201 (2.5)	1385 (2.3)	2120 (4.3)	1700 (2.5)	1146 (2.5)
Infections and infestations	321 (4.0)	3490 (5.7)	3663 (7.4)	3569 (5.3)	2248 (4.9)
Injury, poisoning and procedural complications	411 (5.2)	8138 (13.3)	4825 (9.7)	6110 (9.1)	2668 (5.8)
Investigations	602 (7.6)	4901 (8.0)	5606 (11.3)	5737 (8.5)	2798 (6.1)
Metabolism and nutrition disorders	413 (5.2)	4498 (7.3)	4012 (8.1)	5516 (8.2)	2514 (5.5)
Musculoskeletal and connective tissue disorders	463 (5.8)	5689 (9.3)	4551 (9.1)	5261 (7.8)	2248 (4.9)
Neoplasms benign, malignant and unspecified (incl cysts and polyps)	122 (1.5)	3213 (5.2)	1803 (3.6)	2548 (3.8)	1467 (3.2)
Nervous system disorders	712 (8.9)	6551 (10.7)	5825 (11.7)	7043 (10.5)	3062 (6.7)
Pregnancy, puerperium and perinatal conditions	26 (0.3)	178 (0.3)	599 (1.2)	246 (0.4)	137 (0.3)
Product issues	64 (0.8)	599 (1.0)	507 (1.0)	837 (1.2)	233 (0.5)
Psychiatric disorders	369 (4.6)	5066 (8.3)	4235 (8.5)	5776 (8.6)	2434 (5.3)
Renal and urinary disorders	3473 (43.6)	32,762 (53.4)	23,120 (46.5)	34,365 (51.1)	28,675 (62.6)
Reproductive system and breast disorders	71 (0.9)	663 (1.1)	414 (0.8)	732 (1.1)	352 (0.8)
Respiratory, thoracic and mediastinal disorders	537 (6.7)	4417 (7.2)	3908 (7.9)	4316 (6.4)	2376 (5.2)
Skin and subcutaneous tissue disorders	1073 (13.5)	5303 (8.6)	6655 (13.4)	6445 (9.6)	4315 (9.4)
Social circumstances	21 (0.3)	516 (0.8)	482 (1.0)	439 (0.7)	206 (0.4)
Surgical and medical procedures	68 (0.9)	1021 (1.7)	1125 (2.3)	1246 (1.9)	1328 (2.9)
Vascular disorders	242 (3.0)	2765 (4.5)	2548 (5.1)	2777 (4.1)	1395 (3.0)

Values are presented as *n* (% of substance-specific ICSRs). Percentages are not mutually exclusive and may sum to more than 100% because an individual ICSR may include reactions assigned to more than one SOC.

**Table 3:** System Organ Class (SOC)-level associations meeting the study-defined consistency criterion (all-case RORs with 95% CIs and FDR-adjusted q-values).

System Organ Class	RAB *n*	vs. ESO	vs. PAN	vs. OME	vs. LAN
Hepatobiliary disorders	465	1.88 (1.69–2.08) q < 0.001	1.29 (1.16–1.43) q < 0.001	1.87 (1.69–2.07) q < 0.001	1.96 (1.76–2.18) q < 0.001
Cardiac disorders	261	0.57 (0.50–0.65) q < 0.001	0.57 (0.50–0.65) q < 0.001	0.65 (0.57–0.74) q < 0.001	0.86 (0.75–0.98) q = 0.029
Endocrine disorders	120	0.47 (0.39–0.56) q < 0.001	0.65 (0.54–0.79) q < 0.001	0.53 (0.44–0.64) q < 0.001	0.66 (0.55–0.80) q < 0.001
Gastrointestinal disorders	1349	0.59 (0.56–0.63) q < 0.001	0.62 (0.58–0.66) q < 0.001	0.64 (0.61–0.68) q < 0.001	0.70 (0.65–0.74) q < 0.001
Infections and infestations	321	0.70 (0.62–0.78) q < 0.001	0.53 (0.47–0.59) q < 0.001	0.75 (0.67–0.84) q < 0.001	0.81 (0.72–0.92) q = 0.001
Neoplasms	122	0.28 (0.23–0.34) q < 0.001	0.41 (0.34–0.50) q < 0.001	0.40 (0.33–0.47) q < 0.001	0.47 (0.39–0.57) q < 0.001
Psychiatric disorders	369	0.54 (0.48–0.60) q < 0.001	0.52 (0.47–0.58) q < 0.001	0.52 (0.46–0.58) q < 0.001	0.87 (0.77–0.97) q = 0.016
Renal and urinary disorders	3473	0.67 (0.64–0.71) q < 0.001	0.89 (0.85–0.93) q < 0.001	0.74 (0.71–0.78) q < 0.001	0.46 (0.44–0.48) q < 0.001
Surgical and medical procedures ^a^	68	0.51 (0.40–0.65) q < 0.001	0.37 (0.29–0.48) q < 0.001	0.46 (0.36–0.58) q < 0.001	0.29 (0.23–0.37) q < 0.001

^a^ Procedural MedDRA SOC, not a clinically interpretable reaction category. Associations had to be FDR-significant (Benjamini–Hochberg over 108 tests, q < 0.05) in the same direction in all four comparisons and to keep that direction with a nominal 95% CI excluding 1 in all four serious-only comparisons. Bonferroni, *p* < 0.05/108 in all four comparisons. RAB *n*, rabeprazole ICSRs within the SOC; ESO, esomeprazole; PAN, pantoprazole; OME, omeprazole; LAN, lansoprazole; ROR, reporting odds ratio; CI, confidence interval.

**Table 4:** Robustness of selected SOC-level reporting patterns in serious-only and HCP-restricted analyses.

System Organ Class	All-Case ROR Range	Serious-Only ROR Range	HCP-Restricted ROR Range	HCP q < 0.05, *n*/4	Pattern in HCP Analysis
Hepatobiliary disorders	**1.29–1.96**	**1.19–1.97**	**1.26–1.79**	**4/4**	Preserved
Skin and subcutaneous tissue disorders ^a^	1.01–1.65	**1.14–1.83**	1.07–1.57	3/4	Preserved for 3/4 comparisons
Cardiac disorders	0.57–0.86	0.53–0.82	0.57–0.84	4/4	Preserved
Endocrine disorders	0.47–0.66	0.48–0.71	0.45–0.88	2/4	Direction preserved but attenuated
Gastrointestinal disorders	0.59–0.70	0.56–0.65	0.83–1.04	2/4	Markedly attenuated
Infections and infestations	0.53–0.81	0.50–0.82	0.44–0.73	4/4	Preserved
Neoplasms benign, malignant and unspecified	0.28–0.47	0.27–0.46	0.44–0.55	4/4	Preserved
Psychiatric disorders	0.52–0.87	0.49–0.82	0.55–1.18	4/4 ^b^	Not directionally consistent
Renal and urinary disorders	0.46–0.89	0.49–0.85	0.26–0.49	4/4	Preserved, with greater magnitude
Surgical and medical procedures ^c^	0.29–0.51	0.30–0.52	0.18–0.27	4/4	Preserved, with greater magnitude

RORs compare rabeprazole with each of the four comparator PPIs. Ranges represent the minimum and maximum ROR across comparisons with esomeprazole, pantoprazole, omeprazole, and lansoprazole. The serious-only analysis was restricted to ICSRs classified as serious. The HCP-restricted analysis included only ICSRs reported by healthcare professionals and was exploratory; q-values were adjusted by the Benjamini–Hochberg procedure across its separate family of 108 tests. HCP q < 0.05, *n*/4 indicates the number of comparator-specific associations meeting the FDR criterion. ^a^ Skin and subcutaneous tissue disorders did not meet the study-defined consistency criterion in the all-case analysis because the comparison with pantoprazole was neutral; it is included because of its consistent positive pattern in the serious-only analysis. ^b^ For Psychiatric disorders, all four HCP-restricted comparisons were FDR-significant, but the direction was reversed versus lansoprazole (ROR 1.18). ^c^ Procedural MedDRA SOC; not a clinically interpretable reaction category. HCP, healthcare professional; ROR, reporting odds ratio; SOC, MedDRA System Organ Class. Bold values indicate statistically significant results (*p* < 0.05).

**Table 5:** Cross-source contextualization of the main rabeprazole disproportionality findings identified in EudraVigilance.

System Organ Class	EudraVigilance Finding	Product Information	Published Pharmacovigilance Evidence
Hepatobiliary disorders	Higher reporting odds for rabeprazole versus all four comparators (ROR 1.29–1.96); pattern persisted in serious-only, sex-stratified, and HCP-restricted analyses	Increased hepatic enzymes, hepatitis, and rare hepatic failure are described for rabeprazole [4]	FAERS analyses have reported disproportionality for hepatobiliary events with rabeprazole, including cholestatic hepatitis, cholestasis, and fulminant hepatitis [30]
Skin and subcutaneous tissue disorders	Higher reporting odds versus esomeprazole, omeprazole, and lansoprazole in the all-case analysis; higher reporting odds versus all four comparators in the serious-only analysis	Rash, pruritus, urticaria, subacute cutaneous lupus erythematosus, and severe cutaneous reactions are described in product information [4]	Severe cutaneous adverse reactions associated with PPIs have been reported in pharmacovigilance and dermatologic studies [31,40]
Renal and urinary disorders	Consistently lower reporting odds for rabeprazole in the all-case analysis; lower reporting persisted in the HCP-restricted analysis	Acute tubulointerstitial nephritis and acute kidney injury are recognized renal adverse reactions associated with PPIs	Pharmacovigilance and observational studies have reported associations between PPIs and acute kidney injury or tubulointerstitial nephritis [12,16,17,32]
Gastrointestinal disorders	Consistently lower reporting odds in the all-case analysis; pattern was attenuated in the HCP-restricted analysis	Diarrhoea, nausea, abdominal pain, constipation, and flatulence are commonly listed gastrointestinal adverse reactions	Gastrointestinal adverse reactions are well documented across the PPI class [1,4,35]
Neoplasms benign, malignant and unspecified	Consistently lower reporting odds for rabeprazole	Long-term gastric enterochromaffin-like cell changes have historically been monitored during PPI therapy	Pharmacovigilance studies have examined neoplasm-related reporting with PPIs [33]

The table provides a descriptive comparison between the present EudraVigilance findings and information from product labeling and published studies. External-source findings were not used in the statistical analysis or in the study-defined consistency criterion. HCP, healthcare professional; ROR, reporting odds ratio.

## Data Availability

The original data presented in the study are openly available in the European database of suspected adverse drug reaction reports at adrreports.eu.

## Supplementary Materials

The following supporting information can be downloaded at: https://www.mdpi.com/article/10.3390/jox16060200/s1. Table S1: All-case pairwise RORs across the 27 SOCs (primary analysis; 108 tests); Table S2: Serious-only pairwise RORs across the 27 SOCs (sensitivity analysis; 108 tests); Table S3: Reporter group, geographic origin, and missing sex and age within each SOC, by PPI; Table S4: Pairwise RORs restricted to reports from healthcare professionals (post hoc analysis); Table S5a: Sex-stratified pairwise reporting odds ratios: female-only and male-only ICSRs; Table S5b: Female/male ratio of reporting odds ratios; Extracted aggregated dataset and analysis scripts. Each ROR table (S1, S2, S4, and S5a) lists the 2 × 2 counts, raw *p*-values, FDR-adjusted q-values, and Bonferroni-adjusted *p*-values for every comparison.

## Abbreviations

The following abbreviations are used in this manuscript:

ADR	Adverse drug reaction
CI	Confidence interval
CYP	Cytochrome P450
CYP2C19	Cytochrome P450 2C19
DILI	Drug-induced liver injury
EEA	European Economic Area
EMA	European Medicines Agency
ESO	Esomeprazole
FAERS	FDA Adverse Event Reporting System
FDA	U.S. Food and Drug Administration
FDR	False discovery rate
HCP	Healthcare professional
IC	Information component
ICSR	Individual case safety report
LAN	Lansoprazole
MedDRA	Medical Dictionary for Regulatory Activities
OME	Omeprazole
OR	Odds ratio
PAN	Pantoprazole
PPI	Proton pump inhibitor
PRR	Proportional reporting ratio
PT	Preferred Term
RAB	Rabeprazole
ROR	Reporting odds ratio
SCLE	Subacute cutaneous lupus erythematosus
SOC	System Organ Class
SRS	Spontaneous reporting system
