# Supplementary material for: Comparative Reporting Patterns of Rabeprazole and Other Proton Pump Inhibitors in EudraVigilance: A System Organ Class-Level Disproportionality Analysis
Source: J Xenobiot. 2026 Oct 19;16(6):200. doi: 10.3390/jox16060200 (PMC13706479; doi:10.3390/jox16060200)
Supplement: Supplementary file 1 [file jox-16-00200-s001.zip › jox-4596316-supplementary.pdf]

Supplementary Materials: Comparative Reporting Patterns of Rabeprazole and Other Proton Pump Inhibitors in EudraVigilance: A System Organ Class-Level Disproportionality Analysis

Radu Dumitru Moleriu, Gabriela Ramona Cioacă, Călin Muntean, Adrian-Cosmin Ilie, Daliborca Cristina Vlad, Teodora Piroș and Ruxandra-Cristina Marin

Table S1. All-case pairwise reporting odds ratios, rabeprazole versus each comparator, across the 27 SOCs (primary analysis; 108 tests).

Comparator	System Organ Class	a	b	c	d	ROR (95% CI)	p	q (BH-FDR)	p (Bonferroni)
Esomeprazole	Blood and lymphatic system disorders	679	7,281	5,361	55,956	0.97 (0.90-1.06)	0.526	0.580	1.000
Esomeprazole	Cardiac disorders	261	7,699	3,428	57,889	0.57 (0.50-0.65)	<0.001	<0.001	<0.001
Esomeprazole	Congenital, familial and genetic disorders	18	7,942	226	61,091	0.61 (0.38-0.99)	0.046	0.058	1.000
Esomeprazole	Ear and labyrinth disorders	69	7,891	846	60,471	0.63 (0.49-0.80)	<0.001	<0.001	0.020
Esomeprazole	Endocrine disorders	120	7,840	1,954	59,363	0.47 (0.39-0.56)	<0.001	<0.001	<0.001
Esomeprazole	Eye disorders	170	7,790	1,704	59,613	0.76 (0.65-0.90)	<0.001	0.001	0.098
Esomeprazole	Gastrointestinal disorders	1,349	6,611	15,743	45,574	0.59 (0.56-0.63)	<0.001	<0.001	<0.001
Esomeprazole	General disorders and administration site conditions	1,354	6,606	11,950	49,367	0.85 (0.80-0.90)	<0.001	<0.001	<0.001
Esomeprazole	Hepatobiliary disorders	465	7,495	1,962	59,355	1.88 (1.69-2.08)	<0.001	<0.001	<0.001
Esomeprazole	Immune system disorders	201	7,759	1,385	59,932	1.12 (0.97-1.30)	0.135	0.161	1.000
Esomeprazole	Infections and infestations	321	7,639	3,490	57,827	0.70 (0.62-0.78)	<0.001	<0.001	<0.001
Esomeprazole	Injury, poisoning and procedural complications	411	7,549	8,138	53,179	0.36 (0.32-0.39)	<0.001	<0.001	<0.001
Esomeprazole	Investigations	602	7,358	4,901	56,416	0.94 (0.86-1.03)	0.182	0.209	1.000
Esomeprazole	Metabolism and nutrition disorders	413	7,547	4,498	56,819	0.69 (0.62-0.77)	<0.001	<0.001	<0.001
Esomeprazole	Musculoskeletal and connective tissue disorders	463	7,497	5,689	55,628	0.60 (0.55-0.67)	<0.001	<0.001	<0.001
Esomeprazole	Neoplasms benign, malignant and unspecified (incl cysts and polyps)	122	7,838	3,213	58,104	0.28 (0.23-0.34)	<0.001	<0.001	<0.001
Esomeprazole	Nervous system disorders	712	7,248	6,551	54,766	0.82 (0.76-0.89)	<0.001	<0.001	<0.001
Esomeprazole	Pregnancy, puerperium and perinatal conditions	26	7,934	178	61,139	1.13 (0.75-1.70)	0.574	0.623	1.000
Esomeprazole	Product issues	64	7,896	599	60,718	0.82 (0.63-1.06)	0.137	0.161	1.000
Esomeprazole	Psychiatric disorders	369	7,591	5,066	56,251	0.54 (0.48-0.60)	<0.001	<0.001	<0.001
Esomeprazole	Renal and urinary disorders	3,473	4,487	32,762	28,555	0.67 (0.64-0.71)	<0.001	<0.001	<0.001
Esomeprazole	Reproductive system and breast disorders	71	7,889	663	60,654	0.82 (0.64-1.05)	0.121	0.147	1.000
Esomeprazole	Respiratory, thoracic and mediastinal disorders	537	7,423	4,417	56,900	0.93 (0.85-1.02)	0.136	0.161	1.000
Esomeprazole	Skin and subcutaneous tissue disorders	1,073	6,887	5,303	56,014	1.65 (1.53-1.77)	<0.001	<0.001	<0.001
Esomeprazole	Social circumstances	21	7,939	516	60,801	0.31 (0.20-0.48)	<0.001	<0.001	<0.001
Esomeprazole	Surgical and medical procedures	68	7,892	1,021	60,296	0.51 (0.40-0.65)	<0.001	<0.001	<0.001
Esomeprazole	Vascular disorders	242	7,718	2,765	58,552	0.66 (0.58-0.76)	<0.001	<0.001	<0.001
Pantoprazole	Blood and lymphatic system disorders	679	7,281	4,806	44,939	0.87 (0.80-0.95)	0.001	0.002	0.152
Pantoprazole	Cardiac disorders	261	7,699	2,773	46,972	0.57 (0.50-0.65)	<0.001	<0.001	<0.001
Pantoprazole	Congenital, familial and genetic disorders	18	7,942	317	49,428	0.35 (0.22-0.57)	<0.001	<0.001	0.002

Comparator	System Organ Class	a	b	c	d	ROR (95% CI)	p	q (BH-FDR)	p (Bonferroni)
Pantoprazole	Ear and labyrinth disorders	69	7,891	562	49,183	0.77 (0.60-0.98)	0.037	0.047	1.000
Pantoprazole	Endocrine disorders	120	7,840	1,137	48,608	0.65 (0.54-0.79)	<0.001	<0.001	0.001
Pantoprazole	Eye disorders	170	7,790	1,524	48,221	0.69 (0.59-0.81)	<0.001	<0.001	<0.001
Pantoprazole	Gastrointestinal disorders	1,349	6,611	12,369	37,376	0.62 (0.58-0.66)	<0.001	<0.001	<0.001
Pantoprazole	General disorders and administration site conditions	1,354	6,606	10,476	39,269	0.77 (0.72-0.82)	<0.001	<0.001	<0.001
Pantoprazole	Hepatobiliary disorders	465	7,495	2,286	47,459	1.29 (1.16-1.43)	<0.001	<0.001	<0.001
Pantoprazole	Immune system disorders	201	7,759	2,120	47,625	0.58 (0.50-0.67)	<0.001	<0.001	<0.001
Pantoprazole	Infections and infestations	321	7,639	3,663	46,082	0.53 (0.47-0.59)	<0.001	<0.001	<0.001
Pantoprazole	Injury, poisoning and procedural complications	411	7,549	4,825	44,920	0.51 (0.46-0.56)	<0.001	<0.001	<0.001
Pantoprazole	Investigations	602	7,358	5,606	44,139	0.64 (0.59-0.70)	<0.001	<0.001	<0.001
Pantoprazole	Metabolism and nutrition disorders	413	7,547	4,012	45,733	0.62 (0.56-0.69)	<0.001	<0.001	<0.001
Pantoprazole	Musculoskeletal and connective tissue disorders	463	7,497	4,551	45,194	0.61 (0.56-0.68)	<0.001	<0.001	<0.001
Pantoprazole	Neoplasms benign, malignant and unspecified (incl cysts and polyps)	122	7,838	1,803	47,942	0.41 (0.34-0.50)	<0.001	<0.001	<0.001
Pantoprazole	Nervous system disorders	712	7,248	5,825	43,920	0.74 (0.68-0.80)	<0.001	<0.001	<0.001
Pantoprazole	Pregnancy, puerperium and perinatal conditions	26	7,934	599	49,146	0.27 (0.18-0.40)	<0.001	<0.001	<0.001
Pantoprazole	Product issues	64	7,896	507	49,238	0.79 (0.61-1.02)	0.072	0.090	1.000
Pantoprazole	Psychiatric disorders	369	7,591	4,235	45,510	0.52 (0.47-0.58)	<0.001	<0.001	<0.001
Pantoprazole	Renal and urinary disorders	3,473	4,487	23,120	26,625	0.89 (0.85-0.93)	<0.001	<0.001	<0.001
Pantoprazole	Reproductive system and breast disorders	71	7,889	414	49,331	1.07 (0.83-1.38)	0.588	0.623	1.000
Pantoprazole	Respiratory, thoracic and mediastinal disorders	537	7,423	3,908	45,837	0.85 (0.77-0.93)	<0.001	<0.001	0.062
Pantoprazole	Skin and subcutaneous tissue disorders	1,073	6,887	6,655	43,090	1.01 (0.94-1.08)	0.805	0.828	1.000
Pantoprazole	Social circumstances	21	7,939	482	49,263	0.27 (0.17-0.42)	<0.001	<0.001	<0.001
Pantoprazole	Surgical and medical procedures	68	7,892	1,125	48,620	0.37 (0.29-0.48)	<0.001	<0.001	<0.001
Pantoprazole	Vascular disorders	242	7,718	2,548	47,197	0.58 (0.51-0.66)	<0.001	<0.001	<0.001
Omeprazole	Blood and lymphatic system disorders	679	7,281	5,614	61,617	1.02 (0.94-1.11)	0.584	0.623	1.000
Omeprazole	Cardiac disorders	261	7,699	3,327	63,904	0.65 (0.57-0.74)	<0.001	<0.001	<0.001
Omeprazole	Congenital, familial and genetic disorders	18	7,942	287	66,944	0.53 (0.33-0.85)	0.009	0.012	0.949
Omeprazole	Ear and labyrinth disorders	69	7,891	809	66,422	0.72 (0.56-0.92)	0.009	0.012	0.921
Omeprazole	Endocrine disorders	120	7,840	1,878	65,353	0.53 (0.44-0.64)	<0.001	<0.001	<0.001
Omeprazole	Eye disorders	170	7,790	1,669	65,562	0.86 (0.73-1.01)	0.058	0.073	1.000
Omeprazole	Gastrointestinal disorders	1,349	6,611	16,181	51,050	0.64 (0.61-0.68)	<0.001	<0.001	<0.001
Omeprazole	General disorders and administration site conditions	1,354	6,606	13,096	54,135	0.85 (0.80-0.90)	<0.001	<0.001	<0.001
Omeprazole	Hepatobiliary disorders	465	7,495	2,158	65,073	1.87 (1.69-2.07)	<0.001	<0.001	<0.001
Omeprazole	Immune system disorders	201	7,759	1,700	65,531	1.00 (0.86-1.16)	0.985	0.985	1.000
Omeprazole	Infections and infestations	321	7,639	3,569	63,662	0.75 (0.67-0.84)	<0.001	<0.001	<0.001
Omeprazole	Injury, poisoning and procedural complications	411	7,549	6,110	61,121	0.54 (0.49-0.60)	<0.001	<0.001	<0.001
Omeprazole	Investigations	602	7,358	5,737	61,494	0.88 (0.80-0.96)	0.003	0.005	0.349
Omeprazole	Metabolism and nutrition disorders	413	7,547	5,516	61,715	0.61 (0.55-0.68)	<0.001	<0.001	<0.001
Omeprazole	Musculoskeletal and connective tissue disorders	463	7,497	5,261	61,970	0.73 (0.66-0.80)	<0.001	<0.001	<0.001
Omeprazole	Neoplasms benign, malignant and unspecified (incl cysts and polyps)	122	7,838	2,548	64,683	0.40 (0.33-0.47)	<0.001	<0.001	<0.001
Omeprazole	Nervous system disorders	712	7,248	7,043	60,188	0.84 (0.77-0.91)	<0.001	<0.001	0.002
Omeprazole	Pregnancy, puerperium and perinatal conditions	26	7,934	246	66,985	0.89 (0.60-1.34)	0.581	0.623	1.000

Comparator	System Organ Class	a	b	c	d	ROR (95% CI)	p	q (BH-FDR)	p (Bonferroni)
Omeprazole	Product issues	64	7,896	837	66,394	0.64 (0.50-0.83)	<0.001	0.001	0.075
Omeprazole	Psychiatric disorders	369	7,591	5,776	61,455	0.52 (0.46-0.58)	<0.001	<0.001	<0.001
Omeprazole	Renal and urinary disorders	3,473	4,487	34,365	32,866	0.74 (0.71-0.78)	<0.001	<0.001	<0.001
Omeprazole	Reproductive system and breast disorders	71	7,889	732	66,499	0.82 (0.64-1.04)	0.107	0.131	1.000
Omeprazole	Respiratory, thoracic and mediastinal disorders	537	7,423	4,316	62,915	1.05 (0.96-1.16)	0.262	0.295	1.000
Omeprazole	Skin and subcutaneous tissue disorders	1,073	6,887	6,445	60,786	1.47 (1.37-1.57)	<0.001	<0.001	<0.001
Omeprazole	Social circumstances	21	7,939	439	66,792	0.40 (0.26-0.62)	<0.001	<0.001	0.005
Omeprazole	Surgical and medical procedures	68	7,892	1,246	65,985	0.46 (0.36-0.58)	<0.001	<0.001	<0.001
Omeprazole	Vascular disorders	242	7,718	2,777	64,454	0.73 (0.64-0.83)	<0.001	<0.001	<0.001
Lansoprazole	Blood and lymphatic system disorders	679	7,281	3,984	41,808	0.98 (0.90-1.07)	0.619	0.649	1.000
Lansoprazole	Cardiac disorders	261	7,699	1,743	44,049	0.86 (0.75-0.98)	0.022	0.029	1.000
Lansoprazole	Congenital, familial and genetic disorders	18	7,942	209	45,583	0.49 (0.31-0.80)	0.004	0.006	0.451
Lansoprazole	Ear and labyrinth disorders	69	7,891	329	45,463	1.21 (0.93-1.57)	0.155	0.180	1.000
Lansoprazole	Endocrine disorders	120	7,840	1,035	44,757	0.66 (0.55-0.80)	<0.001	<0.001	0.002
Lansoprazole	Eye disorders	170	7,790	652	45,140	1.51 (1.27-1.79)	<0.001	<0.001	<0.001
Lansoprazole	Gastrointestinal disorders	1,349	6,611	10,392	35,400	0.70 (0.65-0.74)	<0.001	<0.001	<0.001
Lansoprazole	General disorders and administration site conditions	1,354	6,606	6,505	39,287	1.24 (1.16-1.32)	<0.001	<0.001	<0.001
Lansoprazole	Hepatobiliary disorders	465	7,495	1,404	44,388	1.96 (1.76-2.18)	<0.001	<0.001	<0.001
Lansoprazole	Immune system disorders	201	7,759	1,146	44,646	1.01 (0.87-1.17)	0.906	0.923	1.000
Lansoprazole	Infections and infestations	321	7,639	2,248	43,544	0.81 (0.72-0.92)	<0.001	0.001	0.079
Lansoprazole	Injury, poisoning and procedural complications	411	7,549	2,668	43,124	0.88 (0.79-0.98)	0.019	0.025	1.000
Lansoprazole	Investigations	602	7,358	2,798	42,994	1.26 (1.15-1.38)	<0.001	<0.001	<0.001
Lansoprazole	Metabolism and nutrition disorders	413	7,547	2,514	43,278	0.94 (0.85-1.05)	0.274	0.305	1.000
Lansoprazole	Musculoskeletal and connective tissue disorders	463	7,497	2,248	43,544	1.20 (1.08-1.33)	<0.001	0.001	0.070
Lansoprazole	Neoplasms benign, malignant and unspecified (incl cysts and polyps)	122	7,838	1,467	44,325	0.47 (0.39-0.57)	<0.001	<0.001	<0.001
Lansoprazole	Nervous system disorders	712	7,248	3,062	42,730	1.37 (1.26-1.49)	<0.001	<0.001	<0.001
Lansoprazole	Pregnancy, puerperium and perinatal conditions	26	7,934	137	45,655	1.09 (0.72-1.66)	0.681	0.707	1.000
Lansoprazole	Product issues	64	7,896	233	45,559	1.58 (1.20-2.09)	0.001	0.002	0.124
Lansoprazole	Psychiatric disorders	369	7,591	2,434	43,358	0.87 (0.77-0.97)	0.012	0.016	1.000
Lansoprazole	Renal and urinary disorders	3,473	4,487	28,675	17,117	0.46 (0.44-0.48)	<0.001	<0.001	<0.001
Lansoprazole	Reproductive system and breast disorders	71	7,889	352	45,440	1.16 (0.90-1.50)	0.251	0.285	1.000
Lansoprazole	Respiratory, thoracic and mediastinal disorders	537	7,423	2,376	43,416	1.32 (1.20-1.46)	<0.001	<0.001	<0.001
Lansoprazole	Skin and subcutaneous tissue disorders	1,073	6,887	4,315	41,477	1.50 (1.39-1.61)	<0.001	<0.001	<0.001
Lansoprazole	Social circumstances	21	7,939	206	45,586	0.59 (0.37-0.92)	0.020	0.026	1.000
Lansoprazole	Surgical and medical procedures	68	7,892	1,328	44,464	0.29 (0.23-0.37)	<0.001	<0.001	<0.001
Lansoprazole	Vascular disorders	242	7,718	1,395	44,397	1.00 (0.87-1.15)	0.976	0.985	1.000

a, rabeprazole ICSRs reporting the SOC; b, other rabeprazole ICSRs; c, comparator ICSRs reporting the SOC; d, other comparator ICSRs. Denominators (a + b, c + d) are substance-level numbers of individual cases, never SOC column totals, because one ICSR can report reactions in several SOCs. p, two-sided Wald test of ln(ROR); q, Benjamini-Hochberg false discovery rate-adjusted p-value over the 108 tests of the analysis family; p (Bonferroni), p × 108 (capped at 1); exact p-values are given in the accompanying Excel file. BH-FDR, Benjamini-Hochberg false discovery rate; CI, confidence interval; ICSR, individual case safety report; ROR, reporting odds ratio; SOC, MedDRA System Organ Class.

Table S2. Serious-only pairwise reporting odds ratios, rabeprazole versus each comparator, across the 27 SOCs (numerators and denominators restricted to ICSRs classified as serious; separate family of 108 tests).

Comparator	System Organ Class	a	b	c	d	ROR (95% CI)	p	q (BH-FDR)	p (Bonferroni)
Esomeprazole	Blood and lymphatic system disorders	602	6,307	5,272	50,200	0.91 (0.83-0.99)	0.034	0.042	1.000
Esomeprazole	Cardiac disorders	224	6,685	3,312	52,160	0.53 (0.46-0.61)	<0.001	<0.001	<0.001
Esomeprazole	Congenital, familial and genetic disorders	17	6,892	225	55,247	0.61 (0.37-0.99)	0.046	0.057	1.000
Esomeprazole	Ear and labyrinth disorders	42	6,867	671	54,801	0.50 (0.37-0.68)	<0.001	<0.001	0.001
Esomeprazole	Endocrine disorders	119	6,790	1,936	53,536	0.48 (0.40-0.58)	<0.001	<0.001	<0.001
Esomeprazole	Eye disorders	117	6,792	1,483	53,989	0.63 (0.52-0.76)	<0.001	<0.001	<0.001
Esomeprazole	Gastrointestinal disorders	1,039	5,870	13,387	42,085	0.56 (0.52-0.60)	<0.001	<0.001	<0.001
Esomeprazole	General disorders and administration site conditions	1,108	5,801	10,334	45,138	0.83 (0.78-0.89)	<0.001	<0.001	<0.001
Esomeprazole	Hepatobiliary disorders	416	6,493	1,918	53,554	1.79 (1.60-2.00)	<0.001	<0.001	<0.001
Esomeprazole	Immune system disorders	189	6,720	1,284	54,188	1.19 (1.02-1.39)	0.030	0.038	1.000
Esomeprazole	Infections and infestations	294	6,615	3,370	52,102	0.69 (0.61-0.78)	<0.001	<0.001	<0.001
Esomeprazole	Injury, poisoning and procedural complications	371	6,538	7,584	47,888	0.36 (0.32-0.40)	<0.001	<0.001	<0.001
Esomeprazole	Investigations	539	6,370	4,548	50,924	0.95 (0.86-1.04)	0.255	0.290	1.000
Esomeprazole	Metabolism and nutrition disorders	383	6,526	4,251	51,221	0.71 (0.63-0.79)	<0.001	<0.001	<0.001
Esomeprazole	Musculoskeletal and connective tissue disorders	361	6,548	5,117	50,355	0.54 (0.49-0.61)	<0.001	<0.001	<0.001
Esomeprazole	Neoplasms benign, malignant and unspecified (incl cysts and polyps)	111	6,798	3,210	52,262	0.27 (0.22-0.32)	<0.001	<0.001	<0.001
Esomeprazole	Nervous system disorders	543	6,366	5,449	50,023	0.78 (0.71-0.86)	<0.001	<0.001	<0.001
Esomeprazole	Pregnancy, puerperium and perinatal conditions	25	6,884	162	55,310	1.24 (0.81-1.89)	0.318	0.350	1.000
Esomeprazole	Product issues	33	6,876	322	55,150	0.82 (0.57-1.18)	0.285	0.320	1.000
Esomeprazole	Psychiatric disorders	305	6,604	4,525	50,947	0.52 (0.46-0.59)	<0.001	<0.001	<0.001
Esomeprazole	Renal and urinary disorders	3,440	3,469	32,655	22,817	0.69 (0.66-0.73)	<0.001	<0.001	<0.001
Esomeprazole	Reproductive system and breast disorders	46	6,863	530	54,942	0.69 (0.51-0.94)	0.018	0.023	1.000
Esomeprazole	Respiratory, thoracic and mediastinal disorders	444	6,465	4,023	51,449	0.88 (0.79-0.97)	0.012	0.016	1.000
Esomeprazole	Skin and subcutaneous tissue disorders	873	6,036	4,057	51,415	1.83 (1.70-1.98)	<0.001	<0.001	<0.001
Esomeprazole	Social circumstances	21	6,888	491	54,981	0.34 (0.22-0.53)	<0.001	<0.001	<0.001
Esomeprazole	Surgical and medical procedures	65	6,844	993	54,479	0.52 (0.40-0.67)	<0.001	<0.001	<0.001
Esomeprazole	Vascular disorders	214	6,695	2,646	52,826	0.64 (0.55-0.74)	<0.001	<0.001	<0.001
Pantoprazole	Blood and lymphatic system disorders	602	6,307	4,586	38,085	0.79 (0.73-0.87)	<0.001	<0.001	<0.001
Pantoprazole	Cardiac disorders	224	6,685	2,544	40,127	0.53 (0.46-0.61)	<0.001	<0.001	<0.001
Pantoprazole	Congenital, familial and genetic disorders	17	6,892	313	42,358	0.33 (0.20-0.54)	<0.001	<0.001	0.001
Pantoprazole	Ear and labyrinth disorders	42	6,867	390	42,281	0.66 (0.48-0.91)	0.012	0.015	1.000
Pantoprazole	Endocrine disorders	119	6,790	1,126	41,545	0.65 (0.53-0.78)	<0.001	<0.001	<0.001
Pantoprazole	Eye disorders	117	6,792	1,165	41,506	0.61 (0.51-0.74)	<0.001	<0.001	<0.001
Pantoprazole	Gastrointestinal disorders	1,039	5,870	9,926	32,745	0.58 (0.54-0.63)	<0.001	<0.001	<0.001
Pantoprazole	General disorders and administration site conditions	1,108	5,801	8,811	33,860	0.73 (0.69-0.79)	<0.001	<0.001	<0.001
Pantoprazole	Hepatobiliary disorders	416	6,493	2,177	40,494	1.19 (1.07-1.33)	0.001	0.002	0.159
Pantoprazole	Immune system disorders	189	6,720	1,977	40,694	0.58 (0.50-0.67)	<0.001	<0.001	<0.001
Pantoprazole	Infections and infestations	294	6,615	3,494	39,177	0.50 (0.44-0.56)	<0.001	<0.001	<0.001
Pantoprazole	Injury, poisoning and procedural complications	371	6,538	4,439	38,232	0.49 (0.44-0.55)	<0.001	<0.001	<0.001
Pantoprazole	Investigations	539	6,370	5,008	37,663	0.64 (0.58-0.70)	<0.001	<0.001	<0.001

Comparator	System Organ Class	a	b	c	d	ROR (95% CI)	p	q (BH-FDR)	p (Bonferroni)
Pantoprazole	Metabolism and nutrition disorders	383	6,526	3,670	39,001	0.62 (0.56-0.70)	<0.001	<0.001	<0.001
Pantoprazole	Musculoskeletal and connective tissue disorders	361	6,548	3,827	38,844	0.56 (0.50-0.63)	<0.001	<0.001	<0.001
Pantoprazole	Neoplasms benign, malignant and unspecified (incl cysts and polyps)	111	6,798	1,797	40,874	0.37 (0.31-0.45)	<0.001	<0.001	<0.001
Pantoprazole	Nervous system disorders	543	6,366	4,410	38,261	0.74 (0.67-0.81)	<0.001	<0.001	<0.001
Pantoprazole	Pregnancy, puerperium and perinatal conditions	25	6,884	593	42,078	0.26 (0.17-0.38)	<0.001	<0.001	<0.001
Pantoprazole	Product issues	33	6,876	319	42,352	0.64 (0.44-0.91)	0.014	0.018	1.000
Pantoprazole	Psychiatric disorders	305	6,604	3,674	38,997	0.49 (0.43-0.55)	<0.001	<0.001	<0.001
Pantoprazole	Renal and urinary disorders	3,440	3,469	22,978	19,693	0.85 (0.81-0.89)	<0.001	<0.001	<0.001
Pantoprazole	Reproductive system and breast disorders	46	6,863	251	42,420	1.13 (0.83-1.55)	0.438	0.474	1.000
Pantoprazole	Respiratory, thoracic and mediastinal disorders	444	6,465	3,422	39,249	0.79 (0.71-0.87)	<0.001	<0.001	<0.001
Pantoprazole	Skin and subcutaneous tissue disorders	873	6,036	4,808	37,863	1.14 (1.05-1.23)	<0.001	0.001	0.101
Pantoprazole	Social circumstances	21	6,888	469	42,202	0.27 (0.18-0.43)	<0.001	<0.001	<0.001
Pantoprazole	Surgical and medical procedures	65	6,844	1,106	41,565	0.36 (0.28-0.46)	<0.001	<0.001	<0.001
Pantoprazole	Vascular disorders	214	6,695	2,359	40,312	0.55 (0.47-0.63)	<0.001	<0.001	<0.001
Omeprazole	Blood and lymphatic system disorders	602	6,307	5,490	54,214	0.94 (0.86-1.03)	0.188	0.219	1.000
Omeprazole	Cardiac disorders	224	6,685	3,163	56,541	0.60 (0.52-0.69)	<0.001	<0.001	<0.001
Omeprazole	Congenital, familial and genetic disorders	17	6,892	284	59,420	0.52 (0.32-0.84)	0.008	0.011	0.880
Omeprazole	Ear and labyrinth disorders	42	6,867	603	59,101	0.60 (0.44-0.82)	0.001	0.002	0.150
Omeprazole	Endocrine disorders	119	6,790	1,866	57,838	0.54 (0.45-0.65)	<0.001	<0.001	<0.001
Omeprazole	Eye disorders	117	6,792	1,306	58,398	0.77 (0.64-0.93)	0.007	0.010	0.792
Omeprazole	Gastrointestinal disorders	1,039	5,870	13,357	46,347	0.61 (0.57-0.66)	<0.001	<0.001	<0.001
Omeprazole	General disorders and administration site conditions	1,108	5,801	11,099	48,605	0.84 (0.78-0.89)	<0.001	<0.001	<0.001
Omeprazole	Hepatobiliary disorders	416	6,493	2,089	57,615	1.77 (1.59-1.97)	<0.001	<0.001	<0.001
Omeprazole	Immune system disorders	189	6,720	1,557	58,147	1.05 (0.90-1.22)	0.529	0.561	1.000
Omeprazole	Infections and infestations	294	6,615	3,435	56,269	0.73 (0.64-0.82)	<0.001	<0.001	<0.001
Omeprazole	Injury, poisoning and procedural complications	371	6,538	5,620	54,084	0.55 (0.49-0.61)	<0.001	<0.001	<0.001
Omeprazole	Investigations	539	6,370	5,259	54,445	0.88 (0.80-0.96)	0.005	0.007	0.536
Omeprazole	Metabolism and nutrition disorders	383	6,526	5,132	54,572	0.62 (0.56-0.69)	<0.001	<0.001	<0.001
Omeprazole	Musculoskeletal and connective tissue disorders	361	6,548	4,643	55,061	0.65 (0.59-0.73)	<0.001	<0.001	<0.001
Omeprazole	Neoplasms benign, malignant and unspecified (incl cysts and polyps)	111	6,798	2,538	57,166	0.37 (0.30-0.45)	<0.001	<0.001	<0.001
Omeprazole	Nervous system disorders	543	6,366	5,676	54,028	0.81 (0.74-0.89)	<0.001	<0.001	<0.001
Omeprazole	Pregnancy, puerperium and perinatal conditions	25	6,884	232	59,472	0.93 (0.62-1.41)	0.734	0.770	1.000
Omeprazole	Product issues	33	6,876	456	59,248	0.62 (0.44-0.89)	0.009	0.012	0.968
Omeprazole	Psychiatric disorders	305	6,604	5,160	54,544	0.49 (0.43-0.55)	<0.001	<0.001	<0.001
Omeprazole	Renal and urinary disorders	3,440	3,469	34,219	25,485	0.74 (0.70-0.78)	<0.001	<0.001	<0.001
Omeprazole	Reproductive system and breast disorders	46	6,863	516	59,188	0.77 (0.57-1.04)	0.089	0.105	1.000
Omeprazole	Respiratory, thoracic and mediastinal disorders	444	6,465	3,845	55,859	1.00 (0.90-1.10)	0.965	0.983	1.000
Omeprazole	Skin and subcutaneous tissue disorders	873	6,036	4,802	54,902	1.65 (1.53-1.79)	<0.001	<0.001	<0.001
Omeprazole	Social circumstances	21	6,888	428	59,276	0.42 (0.27-0.65)	<0.001	<0.001	0.013
Omeprazole	Surgical and medical procedures	65	6,844	1,230	58,474	0.45 (0.35-0.58)	<0.001	<0.001	<0.001
Omeprazole	Vascular disorders	214	6,695	2,609	57,095	0.70 (0.61-0.81)	<0.001	<0.001	<0.001
Lansoprazole	Blood and lymphatic system disorders	602	6,307	3,855	38,793	0.96 (0.88-1.05)	0.380	0.414	1.000

Comparator	System Organ Class	a	b	c	d	ROR (95% CI)	p	q (BH-FDR)	p (Bonferroni)
Lansoprazole	Cardiac disorders	224	6,685	1,676	40,972	0.82 (0.71-0.94)	0.006	0.008	0.629
Lansoprazole	Congenital, familial and genetic disorders	17	6,892	207	42,441	0.51 (0.31-0.83)	0.007	0.010	0.752
Lansoprazole	Ear and labyrinth disorders	42	6,867	259	42,389	1.00 (0.72-1.39)	0.995	0.995	1.000
Lansoprazole	Endocrine disorders	119	6,790	1,023	41,625	0.71 (0.59-0.86)	<0.001	<0.001	0.058
Lansoprazole	Eye disorders	117	6,792	559	42,089	1.30 (1.06-1.59)	0.011	0.015	1.000
Lansoprazole	Gastrointestinal disorders	1,039	5,870	9,174	33,474	0.65 (0.60-0.69)	<0.001	<0.001	<0.001
Lansoprazole	General disorders and administration site conditions	1,108	5,801	5,967	36,681	1.17 (1.09-1.26)	<0.001	<0.001	<0.001
Lansoprazole	Hepatobiliary disorders	416	6,493	1,341	41,307	1.97 (1.76-2.21)	<0.001	<0.001	<0.001
Lansoprazole	Immune system disorders	189	6,720	1,061	41,587	1.10 (0.94-1.29)	0.223	0.257	1.000
Lansoprazole	Infections and infestations	294	6,615	2,198	40,450	0.82 (0.72-0.93)	0.002	0.002	0.167
Lansoprazole	Injury, poisoning and procedural complications	371	6,538	2,426	40,222	0.94 (0.84-1.05)	0.287	0.320	1.000
Lansoprazole	Investigations	539	6,370	2,613	40,035	1.30 (1.18-1.43)	<0.001	<0.001	<0.001
Lansoprazole	Metabolism and nutrition disorders	383	6,526	2,396	40,252	0.99 (0.88-1.10)	0.803	0.833	1.000
Lansoprazole	Musculoskeletal and connective tissue disorders	361	6,548	2,044	40,604	1.10 (0.98-1.23)	0.121	0.142	1.000
Lansoprazole	Neoplasms benign, malignant and unspecified (incl cysts and polyps)	111	6,798	1,465	41,183	0.46 (0.38-0.56)	<0.001	<0.001	<0.001
Lansoprazole	Nervous system disorders	543	6,366	2,612	40,036	1.31 (1.19-1.44)	<0.001	<0.001	<0.001
Lansoprazole	Pregnancy, puerperium and perinatal conditions	25	6,884	131	42,517	1.18 (0.77-1.81)	0.452	0.483	1.000
Lansoprazole	Product issues	33	6,876	141	42,507	1.45 (0.99-2.12)	0.057	0.069	1.000
Lansoprazole	Psychiatric disorders	305	6,604	2,273	40,375	0.82 (0.73-0.93)	0.002	0.002	0.163
Lansoprazole	Renal and urinary disorders	3,440	3,469	28,622	14,026	0.49 (0.46-0.51)	<0.001	<0.001	<0.001
Lansoprazole	Reproductive system and breast disorders	46	6,863	287	42,361	0.99 (0.72-1.35)	0.946	0.973	1.000
Lansoprazole	Respiratory, thoracic and mediastinal disorders	444	6,465	2,202	40,446	1.26 (1.14-1.40)	<0.001	<0.001	0.002
Lansoprazole	Skin and subcutaneous tissue disorders	873	6,036	3,346	39,302	1.70 (1.57-1.84)	<0.001	<0.001	<0.001
Lansoprazole	Social circumstances	21	6,888	200	42,448	0.65 (0.41-1.02)	0.058	0.070	1.000
Lansoprazole	Surgical and medical procedures	65	6,844	1,320	41,328	0.30 (0.23-0.38)	<0.001	<0.001	<0.001
Lansoprazole	Vascular disorders	214	6,695	1,320	41,328	1.00 (0.86-1.16)	0.992	0.995	1.000

a, rabeprazole ICSRs reporting the SOC; b, other rabeprazole ICSRs; c, comparator ICSRs reporting the SOC; d, other comparator ICSRs. Denominators (a + b, c + d) are substance-level numbers of individual cases, never SOC column totals, because one ICSR can report reactions in several SOCs. p, two-sided Wald test of ln(ROR); q, Benjamini-Hochberg false discovery rate-adjusted p-value over the 108 tests of the analysis family; p (Bonferroni), p × 108 (capped at 1); exact p-values are given in the accompanying Excel file. BH-FDR, Benjamini-Hochberg false discovery rate; CI, confidence interval; ICSR, individual case safety report; ROR, reporting odds ratio; SOC, MedDRA System Organ Class.

Table S3. Reporter group, geographic origin and missing sex and age within each SOC, by PPI.

PPI	System Organ Class	ICSRs	HCP, n (%)	Non-HCP, n	EEA, n	Non-EEA, n (%)	Sex not specified, n (%)	Age not specified, n (%)
Rabeprazole	Blood and lymphatic system disorders	679	521 (76.7)	156	159	520 (76.6)	8 (1.2)	52 (7.7)
Rabeprazole	Cardiac disorders	261	190 (72.8)	70	48	213 (81.6)	9 (3.4)	44 (16.9)
Rabeprazole	Congenital, familial and genetic disorders	18	16 (88.9)	2	4	14 (77.8)	4 (22.2)	8 (44.4)
Rabeprazole	Ear and labyrinth disorders	69	47 (68.1)	22	42	27 (39.1)	2 (2.9)	9 (13.0)
Rabeprazole	Endocrine disorders	120	44 (36.7)	76	12	108 (90.0)	0 (0.0)	8 (6.7)
Rabeprazole	Eye disorders	170	130 (76.5)	39	70	100 (58.8)	2 (1.2)	46 (27.1)
Rabeprazole	Gastrointestinal disorders	1,349	984 (72.9)	362	470	879 (65.2)	26 (1.9)	269 (19.9)
Rabeprazole	General disorders and administration site conditions	1,354	804 (59.4)	544	394	960 (70.9)	267 (19.7)	469 (34.6)
Rabeprazole	Hepatobiliary disorders	465	425 (91.4)	37	75	390 (83.9)	3 (0.6)	21 (4.5)
Rabeprazole	Immune system disorders	201	163 (81.1)	36	53	148 (73.6)	1 (0.5)	21 (10.4)
Rabeprazole	Infections and infestations	321	234 (72.9)	85	66	255 (79.4)	24 (7.5)	62 (19.3)
Rabeprazole	Injury, poisoning and procedural complications	411	312 (75.9)	98	104	307 (74.7)	9 (2.2)	55 (13.4)
Rabeprazole	Investigations	602	508 (84.4)	91	137	465 (77.2)	6 (1.0)	66 (11.0)
Rabeprazole	Metabolism and nutrition disorders	413	350 (84.7)	61	160	253 (61.3)	6 (1.5)	41 (9.9)
Rabeprazole	Musculoskeletal and connective tissue disorders	463	308 (66.5)	153	190	273 (59.0)	12 (2.6)	70 (15.1)
Rabeprazole	Neoplasms benign, malignant and unspecified (incl cysts and polyps)	122	73 (59.8)	49	10	112 (91.8)	1 (0.8)	40 (32.8)
Rabeprazole	Nervous system disorders	712	558 (78.4)	146	283	429 (60.3)	6 (0.8)	105 (14.7)
Rabeprazole	Pregnancy, puerperium and perinatal conditions	26	26 (100.0)	0	14	12 (46.2)	2 (7.7)	8 (30.8)
Rabeprazole	Product issues	64	43 (67.2)	21	46	18 (28.1)	1 (1.6)	17 (26.6)
Rabeprazole	Psychiatric disorders	369	261 (70.7)	106	136	233 (63.1)	15 (4.1)	74 (20.1)
Rabeprazole	Renal and urinary disorders	3,473	775 (22.3)	2,696	94	3,379 (97.3)	1,621 (46.7)	2,207 (63.5)
Rabeprazole	Reproductive system and breast disorders	71	57 (80.3)	14	34	37 (52.1)	1 (1.4)	14 (19.7)
Rabeprazole	Respiratory, thoracic and mediastinal disorders	537	429 (79.9)	107	143	394 (73.4)	10 (1.9)	81 (15.1)
Rabeprazole	Skin and subcutaneous tissue disorders	1,073	976 (91.0)	96	340	733 (68.3)	7 (0.7)	78 (7.3)
Rabeprazole	Social circumstances	21	18 (85.7)	3	6	15 (71.4)	0 (0.0)	5 (23.8)
Rabeprazole	Surgical and medical procedures	68	32 (47.1)	36	8	60 (88.2)	16 (23.5)	45 (66.2)
Rabeprazole	Vascular disorders	242	190 (78.5)	51	69	173 (71.5)	1 (0.4)	46 (19.0)
Esomeprazole	Blood and lymphatic system disorders	5,361	2,910 (54.3)	2,444	1,460	3,901 (72.8)	77 (1.4)	583 (10.9)
Esomeprazole	Cardiac disorders	3,428	1,832 (53.4)	1,595	428	3,000 (87.5)	83 (2.4)	524 (15.3)
Esomeprazole	Congenital, familial and genetic disorders	226	160 (70.8)	66	29	197 (87.2)	15 (6.6)	70 (31.0)
Esomeprazole	Ear and labyrinth disorders	846	438 (51.8)	408	272	574 (67.8)	8 (0.9)	88 (10.4)
Esomeprazole	Endocrine disorders	1,954	581 (29.7)	1,373	98	1,856 (95.0)	20 (1.0)	156 (8.0)
Esomeprazole	Eye disorders	1,704	883 (51.8)	819	439	1,265 (74.2)	16 (0.9)	158 (9.3)
Esomeprazole	Gastrointestinal disorders	15,743	6,168 (39.2)	9,562	3,486	12,257 (77.9)	1,477 (9.4)	3,999 (25.4)
Esomeprazole	General disorders and administration site conditions	11,950	5,253 (44.0)	6,687	2,666	9,284 (77.7)	1,404 (11.7)	3,207 (26.8)
Esomeprazole	Hepatobiliary disorders	1,962	1,602 (81.7)	347	633	1,329 (67.7)	44 (2.2)	178 (9.1)
Esomeprazole	Immune system disorders	1,385	940 (67.9)	444	371	1,014 (73.2)	41 (3.0)	232 (16.8)
Esomeprazole	Infections and infestations	3,490	1,941 (55.6)	1,545	430	3,060 (87.7)	77 (2.2)	548 (15.7)
Esomeprazole	Injury, poisoning and procedural complications	8,138	3,596 (44.2)	4,538	1,002	7,136 (87.7)	224 (2.8)	1,200 (14.7)
Esomeprazole	Investigations	4,901	3,001 (61.2)	1,890	953	3,948 (80.6)	102 (2.1)	685 (14.0)
Esomeprazole	Metabolism and nutrition disorders	4,498	2,927 (65.1)	1,564	1,210	3,288 (73.1)	76 (1.7)	480 (10.7)

PPI	System Organ Class	ICSRs	HCP, n (%)	Non-HCP, n	EEA, n	Non-EEA, n (%)	Sex not specified, n (%)	Age not specified, n (%)
Esomeprazole	Musculoskeletal and connective tissue disorders	5,689	2,940 (51.7)	2,746	989	4,700 (82.6)	85 (1.5)	676 (11.9)
Esomeprazole	Neoplasms benign, malignant and unspecified (incl cysts and polyps)	3,213	975 (30.3)	2,238	71	3,142 (97.8)	217 (6.8)	1,123 (35.0)
Esomeprazole	Nervous system disorders	6,551	3,526 (53.8)	3,013	2,051	4,500 (68.7)	134 (2.0)	888 (13.6)
Esomeprazole	Pregnancy, puerperium and perinatal conditions	178	148 (83.1)	30	69	109 (61.2)	13 (7.3)	55 (30.9)
Esomeprazole	Product issues	599	249 (41.6)	350	328	271 (45.2)	18 (3.0)	172 (28.7)
Esomeprazole	Psychiatric disorders	5,066	2,305 (45.5)	2,756	1,054	4,012 (79.2)	731 (14.4)	1,254 (24.8)
Esomeprazole	Renal and urinary disorders	32,762	9,832 (30.0)	22,925	594	32,168 (98.2)	7,152 (21.8)	16,014 (48.9)
Esomeprazole	Reproductive system and breast disorders	663	440 (66.4)	223	194	469 (70.7)	9 (1.4)	72 (10.9)
Esomeprazole	Respiratory, thoracic and mediastinal disorders	4,417	2,417 (54.7)	1,994	882	3,535 (80.0)	66 (1.5)	564 (12.8)
Esomeprazole	Skin and subcutaneous tissue disorders	5,303	4,166 (78.6)	1,134	2,729	2,574 (48.5)	85 (1.6)	547 (10.3)
Esomeprazole	Social circumstances	516	289 (56.0)	227	46	470 (91.1)	6 (1.2)	90 (17.4)
Esomeprazole	Surgical and medical procedures	1,021	748 (73.3)	272	60	961 (94.1)	65 (6.4)	505 (49.5)
Esomeprazole	Vascular disorders	2,765	1,584 (57.3)	1,178	371	2,394 (86.6)	41 (1.5)	372 (13.5)
Pantoprazole	Blood and lymphatic system disorders	4,806	3,105 (64.6)	1,635	1,902	2,904 (60.4)	68 (1.4)	587 (12.2)
Pantoprazole	Cardiac disorders	2,773	1,904 (68.7)	834	850	1,923 (69.3)	85 (3.1)	619 (22.3)
Pantoprazole	Congenital, familial and genetic disorders	317	289 (91.2)	24	78	239 (75.4)	42 (13.2)	112 (35.3)
Pantoprazole	Ear and labyrinth disorders	562	345 (61.4)	207	297	265 (47.2)	11 (2.0)	101 (18.0)
Pantoprazole	Endocrine disorders	1,137	333 (29.3)	801	143	994 (87.4)	13 (1.1)	105 (9.2)
Pantoprazole	Eye disorders	1,524	1,039 (68.2)	464	667	857 (56.2)	27 (1.8)	323 (21.2)
Pantoprazole	Gastrointestinal disorders	12,369	6,694 (54.1)	5,591	4,264	8,105 (65.5)	880 (7.1)	3,255 (26.3)
Pantoprazole	General disorders and administration site conditions	10,476	6,596 (63.0)	3,779	3,536	6,940 (66.2)	1,023 (9.8)	3,082 (29.4)
Pantoprazole	Hepatobiliary disorders	2,286	2,026 (88.6)	191	954	1,332 (58.3)	42 (1.8)	277 (12.1)
Pantoprazole	Immune system disorders	2,120	1,793 (84.6)	296	593	1,527 (72.0)	69 (3.3)	447 (21.1)
Pantoprazole	Infections and infestations	3,663	2,936 (80.2)	693	754	2,909 (79.4)	91 (2.5)	852 (23.3)
Pantoprazole	Injury, poisoning and procedural complications	4,825	3,796 (78.7)	1,009	1,257	3,568 (73.9)	177 (3.7)	1,173 (24.3)
Pantoprazole	Investigations	5,606	4,460 (79.6)	1,050	1,903	3,703 (66.1)	114 (2.0)	1,016 (18.1)
Pantoprazole	Metabolism and nutrition disorders	4,012	3,233 (80.6)	758	1,653	2,359 (58.8)	85 (2.1)	590 (14.7)
Pantoprazole	Musculoskeletal and connective tissue disorders	4,551	3,101 (68.1)	1,420	1,301	3,250 (71.4)	81 (1.8)	908 (20.0)
Pantoprazole	Neoplasms benign, malignant and unspecified (incl cysts and polyps)	1,803	911 (50.5)	884	154	1,649 (91.5)	120 (6.7)	790 (43.8)
Pantoprazole	Nervous system disorders	5,825	3,885 (66.7)	1,872	2,756	3,069 (52.7)	104 (1.8)	1,118 (19.2)
Pantoprazole	Pregnancy, puerperium and perinatal conditions	599	571 (95.3)	17	83	516 (86.1)	45 (7.5)	144 (24.0)
Pantoprazole	Product issues	507	348 (68.6)	158	226	281 (55.4)	16 (3.2)	123 (24.3)
Pantoprazole	Psychiatric disorders	4,235	2,668 (63.0)	1,529	1,381	2,854 (67.4)	408 (9.6)	989 (23.4)
Pantoprazole	Renal and urinary disorders	23,120	8,029 (34.7)	15,050	903	22,217 (96.1)	4,586 (19.8)	11,243 (48.6)
Pantoprazole	Reproductive system and breast disorders	414	254 (61.4)	158	254	160 (38.6)	7 (1.7)	65 (15.7)
Pantoprazole	Respiratory, thoracic and mediastinal disorders	3,908	2,936 (75.1)	932	1,257	2,650 (67.8)	68 (1.7)	741 (19.0)
Pantoprazole	Skin and subcutaneous tissue disorders	6,655	5,463 (82.1)	1,091	3,897	2,757 (41.4)	114 (1.7)	867 (13.0)
Pantoprazole	Social circumstances	482	378 (78.4)	103	45	437 (90.7)	14 (2.9)	141 (29.3)
Pantoprazole	Surgical and medical procedures	1,125	820 (72.9)	298	91	1,034 (91.9)	62 (5.5)	616 (54.8)
Pantoprazole	Vascular disorders	2,548	1,985 (77.9)	524	756	1,792 (70.3)	67 (2.6)	500 (19.6)
Omeprazole	Blood and lymphatic system disorders	5,614	3,109 (55.4)	2,451	1,131	4,483 (79.9)	102 (1.8)	739 (13.2)
Omeprazole	Cardiac disorders	3,327	2,013 (60.5)	1,293	659	2,668 (80.2)	91 (2.7)	592 (17.8)

PPI	System Organ Class	ICSRs	HCP, n (%)	Non-HCP, n	EEA, n	Non-EEA, n (%)	Sex not specified, n (%)	Age not specified, n (%)
Omeprazole	Congenital, familial and genetic disorders	287	238 (82.9)	45	90	197 (68.6)	40 (13.9)	133 (46.3)
Omeprazole	Ear and labyrinth disorders	809	462 (57.1)	343	347	462 (57.1)	10 (1.2)	136 (16.8)
Omeprazole	Endocrine disorders	1,878	570 (30.4)	1,304	156	1,722 (91.7)	22 (1.2)	150 (8.0)
Omeprazole	Eye disorders	1,669	954 (57.2)	705	683	986 (59.1)	25 (1.5)	234 (14.0)
Omeprazole	Gastrointestinal disorders	16,181	7,141 (44.1)	8,983	4,564	11,617 (71.8)	1,861 (11.5)	4,570 (28.2)
Omeprazole	General disorders and administration site conditions	13,096	6,663 (50.9)	6,351	3,832	9,264 (70.7)	1,923 (14.7)	3,952 (30.2)
Omeprazole	Hepatobiliary disorders	2,158	1,852 (85.8)	267	695	1,463 (67.8)	68 (3.2)	211 (9.8)
Omeprazole	Immune system disorders	1,700	1,272 (74.8)	423	640	1,060 (62.4)	76 (4.5)	372 (21.9)
Omeprazole	Infections and infestations	3,569	2,356 (66.0)	1,192	671	2,898 (81.2)	111 (3.1)	744 (20.8)
Omeprazole	Injury, poisoning and procedural complications	6,110	3,605 (59.0)	2,489	1,320	4,790 (78.4)	181 (3.0)	1,050 (17.2)
Omeprazole	Investigations	5,737	4,001 (69.7)	1,679	1,525	4,212 (73.4)	138 (2.4)	886 (15.4)
Omeprazole	Metabolism and nutrition disorders	5,516	4,173 (75.7)	1,325	2,087	3,429 (62.2)	238 (4.3)	696 (12.6)
Omeprazole	Musculoskeletal and connective tissue disorders	5,261	2,884 (54.8)	2,359	1,349	3,912 (74.4)	102 (1.9)	741 (14.1)
Omeprazole	Neoplasms benign, malignant and unspecified (incl cysts and polyps)	2,548	1,046 (41.1)	1,498	146	2,402 (94.3)	221 (8.7)	1,047 (41.1)
Omeprazole	Nervous system disorders	7,043	4,182 (59.4)	2,811	2,904	4,139 (58.8)	160 (2.3)	1,030 (14.6)
Omeprazole	Pregnancy, puerperium and perinatal conditions	246	218 (88.6)	27	94	152 (61.8)	12 (4.9)	110 (44.7)
Omeprazole	Product issues	837	370 (44.2)	466	458	379 (45.3)	32 (3.8)	276 (33.0)
Omeprazole	Psychiatric disorders	5,776	2,784 (48.2)	2,966	1,468	4,308 (74.6)	1,129 (19.5)	1,702 (29.5)
Omeprazole	Renal and urinary disorders	34,365	11,374 (33.1)	22,960	931	33,434 (97.3)	7,289 (21.2)	16,794 (48.9)
Omeprazole	Reproductive system and breast disorders	732	468 (63.9)	258	348	384 (52.5)	16 (2.2)	106 (14.5)
Omeprazole	Respiratory, thoracic and mediastinal disorders	4,316	2,653 (61.5)	1,625	1,212	3,104 (71.9)	83 (1.9)	622 (14.4)
Omeprazole	Skin and subcutaneous tissue disorders	6,445	5,010 (77.7)	1,380	3,415	3,030 (47.0)	143 (2.2)	728 (11.3)
Omeprazole	Social circumstances	439	275 (62.6)	164	41	398 (90.7)	15 (3.4)	108 (24.6)
Omeprazole	Surgical and medical procedures	1,246	870 (69.8)	370	74	1,172 (94.1)	89 (7.1)	760 (61.0)
Omeprazole	Vascular disorders	2,777	1,763 (63.5)	993	584	2,193 (79.0)	71 (2.6)	445 (16.0)
Lansoprazole	Blood and lymphatic system disorders	3,984	2,412 (60.5)	1,480	818	3,166 (79.5)	68 (1.7)	574 (14.4)
Lansoprazole	Cardiac disorders	1,743	1,150 (66.0)	586	228	1,515 (86.9)	54 (3.1)	375 (21.5)
Lansoprazole	Congenital, familial and genetic disorders	209	188 (90.0)	20	22	187 (89.5)	36 (17.2)	87 (41.6)
Lansoprazole	Ear and labyrinth disorders	329	232 (70.5)	96	111	218 (66.3)	4 (1.2)	36 (10.9)
Lansoprazole	Endocrine disorders	1,035	255 (24.6)	779	54	981 (94.8)	11 (1.1)	104 (10.0)
Lansoprazole	Eye disorders	652	425 (65.2)	221	179	473 (72.5)	17 (2.6)	88 (13.5)
Lansoprazole	Gastrointestinal disorders	10,392	5,541 (53.3)	4,770	2,153	8,239 (79.3)	1,136 (10.9)	2,940 (28.3)
Lansoprazole	General disorders and administration site conditions	6,505	3,199 (49.2)	3,229	1,165	5,340 (82.1)	1,292 (19.9)	2,432 (37.4)
Lansoprazole	Hepatobiliary disorders	1,404	1,266 (90.2)	101	371	1,033 (73.6)	41 (2.9)	109 (7.8)
Lansoprazole	Immune system disorders	1,146	952 (83.1)	180	301	845 (73.7)	49 (4.3)	215 (18.8)
Lansoprazole	Infections and infestations	2,248	1,786 (79.4)	442	250	1,998 (88.9)	85 (3.8)	585 (26.0)
Lansoprazole	Injury, poisoning and procedural complications	2,668	1,687 (63.2)	979	457	2,211 (82.9)	118 (4.4)	614 (23.0)
Lansoprazole	Investigations	2,798	2,283 (81.6)	446	496	2,302 (82.3)	79 (2.8)	516 (18.4)
Lansoprazole	Metabolism and nutrition disorders	2,514	1,978 (78.7)	517	710	1,804 (71.8)	95 (3.8)	349 (13.9)
Lansoprazole	Musculoskeletal and connective tissue disorders	2,248	1,293 (57.5)	943	442	1,806 (80.3)	48 (2.1)	393 (17.5)
Lansoprazole	Neoplasms benign, malignant and unspecified (incl cysts and polyps)	1,467	673 (45.9)	788	45	1,422 (96.9)	135 (9.2)	737 (50.2)
Lansoprazole	Nervous system disorders	3,062	1,984 (64.8)	1,046	921	2,141 (69.9)	74 (2.4)	463 (15.1)

PPI	System Organ Class	ICSRs	HCP, n (%)	Non-HCP, n	EEA, n	Non-EEA, n (%)	Sex not specified, n (%)	Age not specified, n (%)
Lansoprazole	Pregnancy, puerperium and perinatal conditions	137	125 (91.2)	11	28	109 (79.6)	20 (14.6)	37 (27.0)
Lansoprazole	Product issues	233	140 (60.1)	93	123	110 (47.2)	5 (2.1)	77 (33.0)
Lansoprazole	Psychiatric disorders	2,434	1,149 (47.2)	1,278	455	1,979 (81.3)	475 (19.5)	757 (31.1)
Lansoprazole	Renal and urinary disorders	28,675	10,385 (36.2)	18,281	282	28,393 (99.0)	7,276 (25.4)	16,542 (57.7)
Lansoprazole	Reproductive system and breast disorders	352	287 (81.5)	64	110	242 (68.8)	2 (0.6)	33 (9.4)
Lansoprazole	Respiratory, thoracic and mediastinal disorders	2,376	1,740 (73.2)	621	495	1,881 (79.2)	57 (2.4)	410 (17.3)
Lansoprazole	Skin and subcutaneous tissue disorders	4,315	3,715 (86.1)	546	1,905	2,410 (55.9)	86 (2.0)	362 (8.4)
Lansoprazole	Social circumstances	206	152 (73.8)	54	13	193 (93.7)	4 (1.9)	95 (46.1)
Lansoprazole	Surgical and medical procedures	1,328	868 (65.4)	460	22	1,306 (98.3)	306 (23.0)	926 (69.7)
Lansoprazole	Vascular disorders	1,395	1,072 (76.8)	311	234	1,161 (83.2)	28 (2.0)	250 (17.9)

Percentages refer to the ICSRs of the PPI that report the SOC. EEA, European Economic Area (27 EU Member States, Iceland, Liechtenstein and Norway); non-EEA includes all other countries worldwide. HCP, healthcare professional.

Table S4. Pairwise reporting odds ratios restricted to ICSRs reported by healthcare professionals (post hoc analysis).

Comparator	System Organ Class	a	b	c	d	ROR (95% CI)	p	q (BH-FDR)	p (Bonferroni)
Esomeprazole	Blood and lymphatic system disorders	521	4,167	2,910	25,136	1.08 (0.98-1.19)	0.127	0.161	1.000
Esomeprazole	Cardiac disorders	190	4,498	1,832	26,214	0.60 (0.52-0.70)	<0.001	<0.001	<0.001
Esomeprazole	Congenital, familial and genetic disorders	16	4,672	160	27,886	0.60 (0.36-1.00)	0.049	0.071	1.000
Esomeprazole	Ear and labyrinth disorders	47	4,641	438	27,608	0.64 (0.47-0.86)	0.004	0.006	0.392
Esomeprazole	Endocrine disorders	44	4,644	581	27,465	0.45 (0.33-0.61)	<0.001	<0.001	<0.001
Esomeprazole	Eye disorders	130	4,558	883	27,163	0.88 (0.73-1.06)	0.170	0.211	1.000
Esomeprazole	Gastrointestinal disorders	984	3,704	6,168	21,878	0.94 (0.87-1.02)	0.124	0.160	1.000
Esomeprazole	General disorders and administration site conditions	804	3,884	5,253	22,793	0.90 (0.83-0.97)	0.010	0.015	1.000
Esomeprazole	Hepatobiliary disorders	425	4,263	1,602	26,444	1.65 (1.47-1.84)	<0.001	<0.001	<0.001
Esomeprazole	Immune system disorders	163	4,525	940	27,106	1.04 (0.88-1.23)	0.660	0.720	1.000
Esomeprazole	Infections and infestations	234	4,454	1,941	26,105	0.71 (0.61-0.81)	<0.001	<0.001	<0.001
Esomeprazole	Injury, poisoning and procedural complications	312	4,376	3,596	24,450	0.48 (0.43-0.55)	<0.001	<0.001	<0.001
Esomeprazole	Investigations	508	4,180	3,001	25,045	1.01 (0.92-1.12)	0.781	0.835	1.000
Esomeprazole	Metabolism and nutrition disorders	350	4,338	2,927	25,119	0.69 (0.62-0.78)	<0.001	<0.001	<0.001
Esomeprazole	Musculoskeletal and connective tissue disorders	308	4,380	2,940	25,106	0.60 (0.53-0.68)	<0.001	<0.001	<0.001
Esomeprazole	Neoplasms benign, malignant and unspecified (incl cysts and polyps)	73	4,615	975	27,071	0.44 (0.35-0.56)	<0.001	<0.001	<0.001
Esomeprazole	Nervous system disorders	558	4,130	3,526	24,520	0.94 (0.85-1.03)	0.199	0.244	1.000
Esomeprazole	Pregnancy, puerperium and perinatal conditions	26	4,662	148	27,898	1.05 (0.69-1.60)	0.815	0.854	1.000
Esomeprazole	Product issues	43	4,645	249	27,797	1.03 (0.75-1.43)	0.843	0.875	1.000
Esomeprazole	Psychiatric disorders	261	4,427	2,305	25,741	0.66 (0.58-0.75)	<0.001	<0.001	<0.001
Esomeprazole	Renal and urinary disorders	775	3,913	9,832	18,214	0.37 (0.34-0.40)	<0.001	<0.001	<0.001
Esomeprazole	Reproductive system and breast disorders	57	4,631	440	27,606	0.77 (0.59-1.02)	0.068	0.094	1.000
Esomeprazole	Respiratory, thoracic and mediastinal disorders	429	4,259	2,417	25,629	1.07 (0.96-1.19)	0.231	0.274	1.000
Esomeprazole	Skin and subcutaneous tissue disorders	976	3,712	4,166	23,880	1.51 (1.39-1.63)	<0.001	<0.001	<0.001
Esomeprazole	Social circumstances	18	4,670	289	27,757	0.37 (0.23-0.60)	<0.001	<0.001	0.005
Esomeprazole	Surgical and medical procedures	32	4,656	748	27,298	0.25 (0.18-0.36)	<0.001	<0.001	<0.001
Esomeprazole	Vascular disorders	190	4,498	1,584	26,462	0.71 (0.61-0.82)	<0.001	<0.001	<0.001
Pantoprazole	Blood and lymphatic system disorders	521	4,167	3,105	24,601	0.99 (0.90-1.09)	0.851	0.875	1.000
Pantoprazole	Cardiac disorders	190	4,498	1,904	25,802	0.57 (0.49-0.67)	<0.001	<0.001	<0.001
Pantoprazole	Congenital, familial and genetic disorders	16	4,672	289	27,417	0.32 (0.20-0.54)	<0.001	<0.001	0.001
Pantoprazole	Ear and labyrinth disorders	47	4,641	345	27,361	0.80 (0.59-1.09)	0.161	0.202	1.000
Pantoprazole	Endocrine disorders	44	4,644	333	27,373	0.78 (0.57-1.07)	0.121	0.157	1.000
Pantoprazole	Eye disorders	130	4,558	1,039	26,667	0.73 (0.61-0.88)	<0.001	0.002	0.103
Pantoprazole	Gastrointestinal disorders	984	3,704	6,694	21,012	0.83 (0.77-0.90)	<0.001	<0.001	<0.001
Pantoprazole	General disorders and administration site conditions	804	3,884	6,596	21,110	0.66 (0.61-0.72)	<0.001	<0.001	<0.001
Pantoprazole	Hepatobiliary disorders	425	4,263	2,026	25,680	1.26 (1.13-1.41)	<0.001	<0.001	0.003
Pantoprazole	Immune system disorders	163	4,525	1,793	25,913	0.52 (0.44-0.61)	<0.001	<0.001	<0.001
Pantoprazole	Infections and infestations	234	4,454	2,936	24,770	0.44 (0.39-0.51)	<0.001	<0.001	<0.001
Pantoprazole	Injury, poisoning and procedural complications	312	4,376	3,796	23,910	0.45 (0.40-0.51)	<0.001	<0.001	<0.001
Pantoprazole	Investigations	508	4,180	4,460	23,246	0.63 (0.57-0.70)	<0.001	<0.001	<0.001
Pantoprazole	Metabolism and nutrition disorders	350	4,338	3,233	24,473	0.61 (0.54-0.69)	<0.001	<0.001	<0.001

Comparator	System Organ Class	a	b	c	d	ROR (95% CI)	p	q (BH-FDR)	p (Bonferroni)
Pantoprazole	Musculoskeletal and connective tissue disorders	308	4,380	3,101	24,605	0.56 (0.49-0.63)	<0.001	<0.001	<0.001
Pantoprazole	Neoplasms benign, malignant and unspecified (incl cysts and polyps)	73	4,615	911	26,795	0.47 (0.37-0.59)	<0.001	<0.001	<0.001
Pantoprazole	Nervous system disorders	558	4,130	3,885	23,821	0.83 (0.75-0.91)	<0.001	<0.001	0.011
Pantoprazole	Pregnancy, puerperium and perinatal conditions	26	4,662	571	27,135	0.27 (0.18-0.39)	<0.001	<0.001	<0.001
Pantoprazole	Product issues	43	4,645	348	27,358	0.73 (0.53-1.00)	0.050	0.072	1.000
Pantoprazole	Psychiatric disorders	261	4,427	2,668	25,038	0.55 (0.49-0.63)	<0.001	<0.001	<0.001
Pantoprazole	Renal and urinary disorders	775	3,913	8,029	19,677	0.49 (0.45-0.53)	<0.001	<0.001	<0.001
Pantoprazole	Reproductive system and breast disorders	57	4,631	254	27,452	1.33 (1.00-1.78)	0.053	0.074	1.000
Pantoprazole	Respiratory, thoracic and mediastinal disorders	429	4,259	2,936	24,770	0.85 (0.76-0.95)	0.003	0.005	0.293
Pantoprazole	Skin and subcutaneous tissue disorders	976	3,712	5,463	22,243	1.07 (0.99-1.16)	0.081	0.109	1.000
Pantoprazole	Social circumstances	18	4,670	378	27,328	0.28 (0.17-0.45)	<0.001	<0.001	<0.001
Pantoprazole	Surgical and medical procedures	32	4,656	820	26,886	0.23 (0.16-0.32)	<0.001	<0.001	<0.001
Pantoprazole	Vascular disorders	190	4,498	1,985	25,721	0.55 (0.47-0.64)	<0.001	<0.001	<0.001
Omeprazole	Blood and lymphatic system disorders	521	4,167	3,109	31,856	1.28 (1.16-1.41)	<0.001	<0.001	<0.001
Omeprazole	Cardiac disorders	190	4,498	2,013	32,952	0.69 (0.59-0.80)	<0.001	<0.001	<0.001
Omeprazole	Congenital, familial and genetic disorders	16	4,672	238	34,727	0.50 (0.30-0.83)	0.007	0.012	0.792
Omeprazole	Ear and labyrinth disorders	47	4,641	462	34,503	0.76 (0.56-1.02)	0.070	0.095	1.000
Omeprazole	Endocrine disorders	44	4,644	570	34,395	0.57 (0.42-0.78)	<0.001	<0.001	0.041
Omeprazole	Eye disorders	130	4,558	954	34,011	1.02 (0.84-1.22)	0.860	0.877	1.000
Omeprazole	Gastrointestinal disorders	984	3,704	7,141	27,824	1.04 (0.96-1.12)	0.367	0.431	1.000
Omeprazole	General disorders and administration site conditions	804	3,884	6,663	28,302	0.88 (0.81-0.95)	0.002	0.003	0.187
Omeprazole	Hepatobiliary disorders	425	4,263	1,852	33,113	1.78 (1.60-1.99)	<0.001	<0.001	<0.001
Omeprazole	Immune system disorders	163	4,525	1,272	33,693	0.95 (0.81-1.13)	0.580	0.639	1.000
Omeprazole	Infections and infestations	234	4,454	2,356	32,609	0.73 (0.63-0.83)	<0.001	<0.001	<0.001
Omeprazole	Injury, poisoning and procedural complications	312	4,376	3,605	31,360	0.62 (0.55-0.70)	<0.001	<0.001	<0.001
Omeprazole	Investigations	508	4,180	4,001	30,964	0.94 (0.85-1.04)	0.219	0.263	1.000
Omeprazole	Metabolism and nutrition disorders	350	4,338	4,173	30,792	0.60 (0.53-0.67)	<0.001	<0.001	<0.001
Omeprazole	Musculoskeletal and connective tissue disorders	308	4,380	2,884	32,081	0.78 (0.69-0.88)	<0.001	<0.001	0.008
Omeprazole	Neoplasms benign, malignant and unspecified (incl cysts and polyps)	73	4,615	1,046	33,919	0.51 (0.40-0.65)	<0.001	<0.001	<0.001
Omeprazole	Nervous system disorders	558	4,130	4,182	30,783	0.99 (0.91-1.09)	0.909	0.909	1.000
Omeprazole	Pregnancy, puerperium and perinatal conditions	26	4,662	218	34,747	0.89 (0.59-1.34)	0.571	0.636	1.000
Omeprazole	Product issues	43	4,645	370	34,595	0.87 (0.63-1.19)	0.372	0.432	1.000
Omeprazole	Psychiatric disorders	261	4,427	2,784	32,181	0.68 (0.60-0.78)	<0.001	<0.001	<0.001
Omeprazole	Renal and urinary disorders	775	3,913	11,374	23,591	0.41 (0.38-0.45)	<0.001	<0.001	<0.001
Omeprazole	Reproductive system and breast disorders	57	4,631	468	34,497	0.91 (0.69-1.20)	0.491	0.552	1.000
Omeprazole	Respiratory, thoracic and mediastinal disorders	429	4,259	2,653	32,312	1.23 (1.10-1.37)	<0.001	<0.001	0.019
Omeprazole	Skin and subcutaneous tissue disorders	976	3,712	5,010	29,955	1.57 (1.46-1.70)	<0.001	<0.001	<0.001
Omeprazole	Social circumstances	18	4,670	275	34,690	0.49 (0.30-0.78)	0.003	0.005	0.335
Omeprazole	Surgical and medical procedures	32	4,656	870	34,095	0.27 (0.19-0.38)	<0.001	<0.001	<0.001
Omeprazole	Vascular disorders	190	4,498	1,763	33,202	0.80 (0.68-0.93)	0.003	0.006	0.362
Lansoprazole	Blood and lymphatic system disorders	521	4,167	2,412	21,648	1.12 (1.02-1.24)	0.024	0.037	1.000
Lansoprazole	Cardiac disorders	190	4,498	1,150	22,910	0.84 (0.72-0.98)	0.031	0.046	1.000

Comparator	System Organ Class	a	b	c	d	ROR (95% CI)	p	q (BH-FDR)	p (Bonferroni)
Lansoprazole	Congenital, familial and genetic disorders	16	4,672	188	23,872	0.43 (0.26-0.73)	0.001	0.003	0.153
Lansoprazole	Ear and labyrinth disorders	47	4,641	232	23,828	1.04 (0.76-1.43)	0.807	0.854	1.000
Lansoprazole	Endocrine disorders	44	4,644	255	23,805	0.88 (0.64-1.22)	0.454	0.516	1.000
Lansoprazole	Eye disorders	130	4,558	425	23,635	1.59 (1.30-1.94)	<0.001	<0.001	<0.001
Lansoprazole	Gastrointestinal disorders	984	3,704	5,541	18,519	0.89 (0.82-0.96)	0.002	0.004	0.248
Lansoprazole	General disorders and administration site conditions	804	3,884	3,199	20,861	1.35 (1.24-1.47)	<0.001	<0.001	<0.001
Lansoprazole	Hepatobiliary disorders	425	4,263	1,266	22,794	1.79 (1.60-2.01)	<0.001	<0.001	<0.001
Lansoprazole	Immune system disorders	163	4,525	952	23,108	0.87 (0.74-1.04)	0.120	0.157	1.000
Lansoprazole	Infections and infestations	234	4,454	1,786	22,274	0.66 (0.57-0.75)	<0.001	<0.001	<0.001
Lansoprazole	Injury, poisoning and procedural complications	312	4,376	1,687	22,373	0.95 (0.83-1.07)	0.380	0.437	1.000
Lansoprazole	Investigations	508	4,180	2,283	21,777	1.16 (1.05-1.28)	0.004	0.007	0.475
Lansoprazole	Metabolism and nutrition disorders	350	4,338	1,978	22,082	0.90 (0.80-1.01)	0.083	0.111	1.000
Lansoprazole	Musculoskeletal and connective tissue disorders	308	4,380	1,293	22,767	1.24 (1.09-1.41)	0.001	0.002	0.120
Lansoprazole	Neoplasms benign, malignant and unspecified (incl cysts and polyps)	73	4,615	673	23,387	0.55 (0.43-0.70)	<0.001	<0.001	<0.001
Lansoprazole	Nervous system disorders	558	4,130	1,984	22,076	1.50 (1.36-1.66)	<0.001	<0.001	<0.001
Lansoprazole	Pregnancy, puerperium and perinatal conditions	26	4,662	125	23,935	1.07 (0.70-1.63)	0.761	0.822	1.000
Lansoprazole	Product issues	43	4,645	140	23,920	1.58 (1.12-2.23)	0.009	0.014	0.954
Lansoprazole	Psychiatric disorders	261	4,427	1,149	22,911	1.18 (1.02-1.35)	0.022	0.033	1.000
Lansoprazole	Renal and urinary disorders	775	3,913	10,385	13,675	0.26 (0.24-0.28)	<0.001	<0.001	<0.001
Lansoprazole	Reproductive system and breast disorders	57	4,631	287	23,773	1.02 (0.77-1.36)	0.895	0.903	1.000
Lansoprazole	Respiratory, thoracic and mediastinal disorders	429	4,259	1,740	22,320	1.29 (1.16-1.44)	<0.001	<0.001	<0.001
Lansoprazole	Skin and subcutaneous tissue disorders	976	3,712	3,715	20,345	1.44 (1.33-1.56)	<0.001	<0.001	<0.001
Lansoprazole	Social circumstances	18	4,670	152	23,908	0.61 (0.37-0.99)	0.045	0.066	1.000
Lansoprazole	Surgical and medical procedures	32	4,656	868	23,192	0.18 (0.13-0.26)	<0.001	<0.001	<0.001
Lansoprazole	Vascular disorders	190	4,498	1,072	22,988	0.91 (0.77-1.06)	0.218	0.263	1.000

a, rabeprazole ICSRs reporting the SOC; b, other rabeprazole ICSRs; c, comparator ICSRs reporting the SOC; d, other comparator ICSRs. Denominators (a + b, c + d) are substance-level numbers of individual cases, never SOC column totals, because one ICSR can report reactions in several SOCs. p, two-sided Wald test of ln(ROR); q, Benjamini-Hochberg false discovery rate-adjusted p-value over the 108 tests of the analysis family; p (Bonferroni), p × 108 (capped at 1); exact p-values are given in the accompanying Excel file. BH-FDR, Benjamini-Hochberg false discovery rate; CI, confidence interval; ICSR, individual case safety report; ROR, reporting odds ratio; SOC, MedDRA System Organ Class. HCP, healthcare professional.

Table S5a. Sex-stratified pairwise reporting odds ratios: female-only and male-only ICSRs (denominators: female or male ICSRs of each substance; separate family of 108 tests per stratum). ICSRs with unspecified sex are not analysed.

Stratum	Comparator	System Organ Class	a	b	c	d	ROR (95% CI)	p	q (BH-FDR)	p (Bonferroni)
Female	Esomeprazole	Blood and lymphatic system disorders	334	3,230	2,839	29,039	1.06 (0.94-1.19)	0.356	0.395	1.000
Female	Esomeprazole	Cardiac disorders	119	3,445	1,949	29,929	0.53 (0.44-0.64)	<0.001	<0.001	<0.001
Female	Esomeprazole	Congenital, familial and genetic disorders	4	3,560	135	31,743	0.26 (0.10-0.71)	0.009	0.013	0.944
Female	Esomeprazole	Ear and labyrinth disorders	45	3,519	499	31,379	0.80 (0.59-1.09)	0.164	0.195	1.000
Female	Esomeprazole	Endocrine disorders	65	3,499	1,174	30,704	0.49 (0.38-0.63)	<0.001	<0.001	<0.001
Female	Esomeprazole	Eye disorders	84	3,480	1,214	30,664	0.61 (0.49-0.76)	<0.001	<0.001	0.002
Female	Esomeprazole	Gastrointestinal disorders	808	2,756	9,249	22,629	0.72 (0.66-0.78)	<0.001	<0.001	<0.001
Female	Esomeprazole	General disorders and administration site conditions	642	2,922	6,739	25,139	0.82 (0.75-0.90)	<0.001	<0.001	0.001
Female	Esomeprazole	Hepatobiliary disorders	230	3,334	1,032	30,846	2.06 (1.78-2.39)	<0.001	<0.001	<0.001
Female	Esomeprazole	Immune system disorders	155	3,409	989	30,889	1.42 (1.19-1.69)	<0.001	<0.001	0.008
Female	Esomeprazole	Infections and infestations	170	3,394	2,287	29,591	0.65 (0.55-0.76)	<0.001	<0.001	<0.001
Female	Esomeprazole	Injury, poisoning and procedural complications	268	3,296	5,385	26,493	0.40 (0.35-0.45)	<0.001	<0.001	<0.001
Female	Esomeprazole	Investigations	302	3,262	2,989	28,889	0.89 (0.79-1.01)	0.078	0.101	1.000
Female	Esomeprazole	Metabolism and nutrition disorders	236	3,328	2,757	29,121	0.75 (0.65-0.86)	<0.001	<0.001	0.004
Female	Esomeprazole	Musculoskeletal and connective tissue disorders	261	3,303	3,731	28,147	0.60 (0.52-0.68)	<0.001	<0.001	<0.001
Female	Esomeprazole	Neoplasms benign, malignant and unspecified (incl cysts and polyps)	62	3,502	1,768	30,110	0.30 (0.23-0.39)	<0.001	<0.001	<0.001
Female	Esomeprazole	Nervous system disorders	422	3,142	4,261	27,617	0.87 (0.78-0.97)	0.011	0.015	1.000
Female	Esomeprazole	Pregnancy, puerperium and perinatal conditions	21	3,543	135	31,743	1.39 (0.88-2.21)	0.158	0.192	1.000
Female	Esomeprazole	Product issues	36	3,528	363	31,515	0.89 (0.63-1.25)	0.490	0.522	1.000
Female	Esomeprazole	Psychiatric disorders	208	3,356	2,837	29,041	0.63 (0.55-0.73)	<0.001	<0.001	<0.001
Female	Esomeprazole	Renal and urinary disorders	1,057	2,507	14,997	16,881	0.47 (0.44-0.51)	<0.001	<0.001	<0.001
Female	Esomeprazole	Reproductive system and breast disorders	34	3,530	366	31,512	0.83 (0.58-1.18)	0.299	0.340	1.000
Female	Esomeprazole	Respiratory, thoracic and mediastinal disorders	286	3,278	2,874	29,004	0.88 (0.78-1.00)	0.049	0.065	1.000
Female	Esomeprazole	Skin and subcutaneous tissue disorders	685	2,879	3,263	28,615	2.09 (1.91-2.28)	<0.001	<0.001	<0.001
Female	Esomeprazole	Social circumstances	13	3,551	316	31,562	0.37 (0.21-0.64)	<0.001	<0.001	0.042
Female	Esomeprazole	Surgical and medical procedures	28	3,536	498	31,380	0.50 (0.34-0.73)	<0.001	<0.001	0.039
Female	Esomeprazole	Vascular disorders	121	3,443	1,680	30,198	0.63 (0.52-0.76)	<0.001	<0.001	<0.001
Female	Pantoprazole	Blood and lymphatic system disorders	334	3,230	2,564	23,765	0.96 (0.85-1.08)	0.487	0.522	1.000
Female	Pantoprazole	Cardiac disorders	119	3,445	1,597	24,732	0.53 (0.44-0.65)	<0.001	<0.001	<0.001
Female	Pantoprazole	Congenital, familial and genetic disorders	4	3,560	191	26,138	0.15 (0.06-0.41)	<0.001	<0.001	0.023
Female	Pantoprazole	Ear and labyrinth disorders	45	3,519	377	25,952	0.88 (0.64-1.20)	0.422	0.460	1.000
Female	Pantoprazole	Endocrine disorders	65	3,499	675	25,654	0.71 (0.55-0.91)	0.008	0.012	0.857
Female	Pantoprazole	Eye disorders	84	3,480	1,077	25,252	0.57 (0.45-0.71)	<0.001	<0.001	<0.001
Female	Pantoprazole	Gastrointestinal disorders	808	2,756	7,544	18,785	0.73 (0.67-0.79)	<0.001	<0.001	<0.001
Female	Pantoprazole	General disorders and administration site conditions	642	2,922	6,183	20,146	0.72 (0.65-0.78)	<0.001	<0.001	<0.001
Female	Pantoprazole	Hepatobiliary disorders	230	3,334	1,406	24,923	1.22 (1.06-1.41)	0.006	0.010	0.667
Female	Pantoprazole	Immune system disorders	155	3,409	1,620	24,709	0.69 (0.59-0.82)	<0.001	<0.001	0.002
Female	Pantoprazole	Infections and infestations	170	3,394	2,666	23,663	0.44 (0.38-0.52)	<0.001	<0.001	<0.001
Female	Pantoprazole	Injury, poisoning and procedural complications	268	3,296	3,459	22,870	0.54 (0.47-0.61)	<0.001	<0.001	<0.001
Female	Pantoprazole	Investigations	302	3,262	3,645	22,684	0.58 (0.51-0.65)	<0.001	<0.001	<0.001

Stratum	Comparator	System Organ Class	a	b	c	d	ROR (95% CI)	p	q (BH-FDR)	p (Bonferroni)
Female	Pantoprazole	Metabolism and nutrition disorders	236	3,328	2,554	23,775	0.66 (0.57-0.76)	<0.001	<0.001	<0.001
Female	Pantoprazole	Musculoskeletal and connective tissue disorders	261	3,303	3,260	23,069	0.56 (0.49-0.64)	<0.001	<0.001	<0.001
Female	Pantoprazole	Neoplasms benign, malignant and unspecified (incl cysts and polyps)	62	3,502	1,058	25,271	0.42 (0.33-0.55)	<0.001	<0.001	<0.001
Female	Pantoprazole	Nervous system disorders	422	3,142	3,898	22,431	0.77 (0.69-0.86)	<0.001	<0.001	<0.001
Female	Pantoprazole	Pregnancy, puerperium and perinatal conditions	21	3,543	524	25,805	0.29 (0.19-0.45)	<0.001	<0.001	<0.001
Female	Pantoprazole	Product issues	36	3,528	360	25,969	0.74 (0.52-1.04)	0.081	0.103	1.000
Female	Pantoprazole	Psychiatric disorders	208	3,356	2,676	23,653	0.55 (0.47-0.63)	<0.001	<0.001	<0.001
Female	Pantoprazole	Renal and urinary disorders	1,057	2,507	10,811	15,518	0.61 (0.56-0.65)	<0.001	<0.001	<0.001
Female	Pantoprazole	Reproductive system and breast disorders	34	3,530	200	26,129	1.26 (0.87-1.81)	0.218	0.253	1.000
Female	Pantoprazole	Respiratory, thoracic and mediastinal disorders	286	3,278	2,605	23,724	0.79 (0.70-0.90)	<0.001	<0.001	0.044
Female	Pantoprazole	Skin and subcutaneous tissue disorders	685	2,879	4,331	21,998	1.21 (1.11-1.32)	<0.001	<0.001	0.004
Female	Pantoprazole	Social circumstances	13	3,551	352	25,977	0.27 (0.16-0.47)	<0.001	<0.001	<0.001
Female	Pantoprazole	Surgical and medical procedures	28	3,536	625	25,704	0.33 (0.22-0.48)	<0.001	<0.001	<0.001
Female	Pantoprazole	Vascular disorders	121	3,443	1,694	24,635	0.51 (0.42-0.62)	<0.001	<0.001	<0.001
Female	Omeprazole	Blood and lymphatic system disorders	334	3,230	2,979	31,134	1.08 (0.96-1.22)	0.200	0.235	1.000
Female	Omeprazole	Cardiac disorders	119	3,445	1,743	32,370	0.64 (0.53-0.77)	<0.001	<0.001	<0.001
Female	Omeprazole	Congenital, familial and genetic disorders	4	3,560	153	33,960	0.25 (0.09-0.67)	0.006	0.010	0.663
Female	Omeprazole	Ear and labyrinth disorders	45	3,519	479	33,634	0.90 (0.66-1.22)	0.493	0.522	1.000
Female	Omeprazole	Endocrine disorders	65	3,499	1,092	33,021	0.56 (0.44-0.72)	<0.001	<0.001	<0.001
Female	Omeprazole	Eye disorders	84	3,480	1,097	33,016	0.73 (0.58-0.91)	0.005	0.008	0.572
Female	Omeprazole	Gastrointestinal disorders	808	2,756	8,928	25,185	0.83 (0.76-0.90)	<0.001	<0.001	<0.001
Female	Omeprazole	General disorders and administration site conditions	642	2,922	6,694	27,419	0.90 (0.82-0.98)	0.021	0.028	1.000
Female	Omeprazole	Hepatobiliary disorders	230	3,334	1,073	33,040	2.12 (1.83-2.46)	<0.001	<0.001	<0.001
Female	Omeprazole	Immune system disorders	155	3,409	1,048	33,065	1.43 (1.21-1.70)	<0.001	<0.001	0.004
Female	Omeprazole	Infections and infestations	170	3,394	2,169	31,944	0.74 (0.63-0.87)	<0.001	<0.001	0.021
Female	Omeprazole	Injury, poisoning and procedural complications	268	3,296	3,862	30,251	0.64 (0.56-0.72)	<0.001	<0.001	<0.001
Female	Omeprazole	Investigations	302	3,262	3,176	30,937	0.90 (0.80-1.02)	0.101	0.127	1.000
Female	Omeprazole	Metabolism and nutrition disorders	236	3,328	3,178	30,935	0.69 (0.60-0.79)	<0.001	<0.001	<0.001
Female	Omeprazole	Musculoskeletal and connective tissue disorders	261	3,303	3,318	30,795	0.73 (0.64-0.84)	<0.001	<0.001	<0.001
Female	Omeprazole	Neoplasms benign, malignant and unspecified (incl cysts and polyps)	62	3,502	1,283	32,830	0.45 (0.35-0.59)	<0.001	<0.001	<0.001
Female	Omeprazole	Nervous system disorders	422	3,142	4,316	29,797	0.93 (0.83-1.03)	0.165	0.195	1.000
Female	Omeprazole	Pregnancy, puerperium and perinatal conditions	21	3,543	195	33,918	1.03 (0.66-1.62)	0.895	0.903	1.000
Female	Omeprazole	Product issues	36	3,528	513	33,600	0.67 (0.48-0.94)	0.020	0.027	1.000
Female	Omeprazole	Psychiatric disorders	208	3,356	2,922	31,191	0.66 (0.57-0.76)	<0.001	<0.001	<0.001
Female	Omeprazole	Renal and urinary disorders	1,057	2,507	15,563	18,550	0.50 (0.47-0.54)	<0.001	<0.001	<0.001
Female	Omeprazole	Reproductive system and breast disorders	34	3,530	340	33,773	0.96 (0.67-1.36)	0.807	0.822	1.000
Female	Omeprazole	Respiratory, thoracic and mediastinal disorders	286	3,278	2,591	31,522	1.06 (0.93-1.21)	0.358	0.395	1.000
Female	Omeprazole	Skin and subcutaneous tissue disorders	685	2,879	3,891	30,222	1.85 (1.69-2.02)	<0.001	<0.001	<0.001
Female	Omeprazole	Social circumstances	13	3,551	233	33,880	0.53 (0.30-0.93)	0.027	0.036	1.000
Female	Omeprazole	Surgical and medical procedures	28	3,536	616	33,497	0.43 (0.29-0.63)	<0.001	<0.001	0.002
Female	Omeprazole	Vascular disorders	121	3,443	1,606	32,507	0.71 (0.59-0.86)	<0.001	<0.001	0.042
Female	Lansoprazole	Blood and lymphatic system disorders	334	3,230	2,032	20,005	1.02 (0.90-1.15)	0.773	0.795	1.000

Stratum	Comparator	System Organ Class	a	b	c	d	ROR (95% CI)	p	q (BH-FDR)	p (Bonferroni)
Female	Lansoprazole	Cardiac disorders	119	3,445	843	21,194	0.87 (0.71-1.06)	0.157	0.192	1.000
Female	Lansoprazole	Congenital, familial and genetic disorders	4	3,560	109	21,928	0.23 (0.08-0.61)	0.004	0.006	0.379
Female	Lansoprazole	Ear and labyrinth disorders	45	3,519	189	21,848	1.48 (1.07-2.05)	0.019	0.027	1.000
Female	Lansoprazole	Endocrine disorders	65	3,499	589	21,448	0.68 (0.52-0.88)	0.003	0.005	0.330
Female	Lansoprazole	Eye disorders	84	3,480	371	21,666	1.41 (1.11-1.79)	0.005	0.008	0.536
Female	Lansoprazole	Gastrointestinal disorders	808	2,756	5,765	16,272	0.83 (0.76-0.90)	<0.001	<0.001	0.001
Female	Lansoprazole	General disorders and administration site conditions	642	2,922	3,064	18,973	1.36 (1.24-1.49)	<0.001	<0.001	<0.001
Female	Lansoprazole	Hepatobiliary disorders	230	3,334	727	21,310	2.02 (1.74-2.36)	<0.001	<0.001	<0.001
Female	Lansoprazole	Immune system disorders	155	3,409	763	21,274	1.27 (1.06-1.51)	0.008	0.012	0.907
Female	Lansoprazole	Infections and infestations	170	3,394	1,298	20,739	0.80 (0.68-0.94)	0.008	0.012	0.835
Female	Lansoprazole	Injury, poisoning and procedural complications	268	3,296	1,652	20,385	1.00 (0.88-1.15)	0.961	0.961	1.000
Female	Lansoprazole	Investigations	302	3,262	1,537	20,500	1.23 (1.09-1.40)	0.001	0.002	0.143
Female	Lansoprazole	Metabolism and nutrition disorders	236	3,328	1,426	20,611	1.02 (0.89-1.18)	0.735	0.763	1.000
Female	Lansoprazole	Musculoskeletal and connective tissue disorders	261	3,303	1,372	20,665	1.19 (1.04-1.37)	0.013	0.018	1.000
Female	Lansoprazole	Neoplasms benign, malignant and unspecified (incl cysts and polyps)	62	3,502	712	21,325	0.53 (0.41-0.69)	<0.001	<0.001	<0.001
Female	Lansoprazole	Nervous system disorders	422	3,142	1,918	20,119	1.41 (1.26-1.58)	<0.001	<0.001	<0.001
Female	Lansoprazole	Pregnancy, puerperium and perinatal conditions	21	3,543	103	21,934	1.26 (0.79-2.02)	0.332	0.374	1.000
Female	Lansoprazole	Product issues	36	3,528	159	21,878	1.40 (0.98-2.02)	0.067	0.088	1.000
Female	Lansoprazole	Psychiatric disorders	208	3,356	1,178	20,859	1.10 (0.94-1.28)	0.230	0.264	1.000
Female	Lansoprazole	Renal and urinary disorders	1,057	2,507	12,447	9,590	0.32 (0.30-0.35)	<0.001	<0.001	<0.001
Female	Lansoprazole	Reproductive system and breast disorders	34	3,530	128	21,909	1.65 (1.13-2.41)	0.010	0.014	1.000
Female	Lansoprazole	Respiratory, thoracic and mediastinal disorders	286	3,278	1,351	20,686	1.34 (1.17-1.53)	<0.001	<0.001	0.002
Female	Lansoprazole	Skin and subcutaneous tissue disorders	685	2,879	2,658	19,379	1.73 (1.58-1.90)	<0.001	<0.001	<0.001
Female	Lansoprazole	Social circumstances	13	3,551	127	21,910	0.63 (0.36-1.12)	0.115	0.143	1.000
Female	Lansoprazole	Surgical and medical procedures	28	3,536	447	21,590	0.38 (0.26-0.56)	<0.001	<0.001	<0.001
Female	Lansoprazole	Vascular disorders	121	3,443	778	21,259	0.96 (0.79-1.17)	0.684	0.717	1.000
Male	Esomeprazole	Blood and lymphatic system disorders	337	2,356	2,445	18,906	1.11 (0.98-1.25)	0.104	0.163	1.000
Male	Esomeprazole	Cardiac disorders	133	2,560	1,396	19,955	0.74 (0.62-0.89)	0.001	0.004	0.151
Male	Esomeprazole	Congenital, familial and genetic disorders	10	2,683	76	21,275	1.04 (0.54-2.02)	0.900	0.959	1.000
Male	Esomeprazole	Ear and labyrinth disorders	22	2,671	339	21,012	0.51 (0.33-0.79)	0.002	0.006	0.253
Male	Esomeprazole	Endocrine disorders	55	2,638	760	20,591	0.56 (0.43-0.74)	<0.001	<0.001	0.006
Male	Esomeprazole	Eye disorders	84	2,609	474	20,877	1.42 (1.12-1.79)	0.004	0.008	0.395
Male	Esomeprazole	Gastrointestinal disorders	515	2,178	5,017	16,334	0.77 (0.70-0.85)	<0.001	<0.001	<0.001
Male	Esomeprazole	General disorders and administration site conditions	445	2,248	3,807	17,544	0.91 (0.82-1.02)	0.094	0.154	1.000
Male	Esomeprazole	Hepatobiliary disorders	232	2,461	886	20,465	2.18 (1.87-2.53)	<0.001	<0.001	<0.001
Male	Esomeprazole	Immune system disorders	45	2,648	355	20,996	1.01 (0.74-1.37)	0.975	0.975	1.000
Male	Esomeprazole	Infections and infestations	127	2,566	1,126	20,225	0.89 (0.74-1.07)	0.220	0.313	1.000
Male	Esomeprazole	Injury, poisoning and procedural complications	134	2,559	2,529	18,822	0.39 (0.33-0.47)	<0.001	<0.001	<0.001
Male	Esomeprazole	Investigations	294	2,399	1,810	19,541	1.32 (1.16-1.51)	<0.001	<0.001	0.003
Male	Esomeprazole	Metabolism and nutrition disorders	171	2,522	1,665	19,686	0.80 (0.68-0.94)	0.008	0.016	0.839
Male	Esomeprazole	Musculoskeletal and connective tissue disorders	190	2,503	1,873	19,478	0.79 (0.68-0.92)	0.003	0.006	0.300
Male	Esomeprazole	Neoplasms benign, malignant and unspecified (incl cysts and polyps)	59	2,634	1,228	20,123	0.37 (0.28-0.48)	<0.001	<0.001	<0.001

Stratum	Comparator	System Organ Class	a	b	c	d	ROR (95% CI)	p	q (BH-FDR)	p (Bonferroni)
Male	Esomeprazole	Nervous system disorders	284	2,409	2,156	19,195	1.05 (0.92-1.20)	0.468	0.575	1.000
Male	Esomeprazole	Pregnancy, puerperium and perinatal conditions	3	2,690	30	21,321	0.79 (0.24-2.60)	0.701	0.797	1.000
Male	Esomeprazole	Product issues	27	2,666	218	21,133	0.98 (0.66-1.47)	0.928	0.959	1.000
Male	Esomeprazole	Psychiatric disorders	146	2,547	1,498	19,853	0.76 (0.64-0.90)	0.002	0.005	0.223
Male	Esomeprazole	Renal and urinary disorders	795	1,898	10,613	10,738	0.42 (0.39-0.46)	<0.001	<0.001	<0.001
Male	Esomeprazole	Reproductive system and breast disorders	36	2,657	288	21,063	0.99 (0.70-1.40)	0.959	0.968	1.000
Male	Esomeprazole	Respiratory, thoracic and mediastinal disorders	241	2,452	1,477	19,874	1.32 (1.15-1.53)	<0.001	<0.001	0.013
Male	Esomeprazole	Skin and subcutaneous tissue disorders	381	2,312	1,955	19,396	1.63 (1.45-1.84)	<0.001	<0.001	<0.001
Male	Esomeprazole	Social circumstances	8	2,685	194	21,157	0.32 (0.16-0.66)	0.002	0.005	0.201
Male	Esomeprazole	Surgical and medical procedures	24	2,669	458	20,893	0.41 (0.27-0.62)	<0.001	<0.001	0.002
Male	Esomeprazole	Vascular disorders	120	2,573	1,044	20,307	0.91 (0.75-1.10)	0.323	0.448	1.000
Male	Pantoprazole	Blood and lymphatic system disorders	337	2,356	2,174	15,750	1.04 (0.92-1.17)	0.569	0.654	1.000
Male	Pantoprazole	Cardiac disorders	133	2,560	1,091	16,833	0.80 (0.67-0.96)	0.019	0.036	1.000
Male	Pantoprazole	Congenital, familial and genetic disorders	10	2,683	84	17,840	0.79 (0.41-1.53)	0.486	0.589	1.000
Male	Pantoprazole	Ear and labyrinth disorders	22	2,671	174	17,750	0.84 (0.54-1.31)	0.444	0.557	1.000
Male	Pantoprazole	Endocrine disorders	55	2,638	449	17,475	0.81 (0.61-1.08)	0.148	0.219	1.000
Male	Pantoprazole	Eye disorders	84	2,609	420	17,504	1.34 (1.06-1.70)	0.015	0.031	1.000
Male	Pantoprazole	Gastrointestinal disorders	515	2,178	3,945	13,979	0.84 (0.76-0.93)	<0.001	0.002	0.076
Male	Pantoprazole	General disorders and administration site conditions	445	2,248	3,270	14,654	0.89 (0.80-0.99)	0.031	0.055	1.000
Male	Pantoprazole	Hepatobiliary disorders	232	2,461	838	17,086	1.92 (1.65-2.24)	<0.001	<0.001	<0.001
Male	Pantoprazole	Immune system disorders	45	2,648	431	17,493	0.69 (0.51-0.94)	0.019	0.036	1.000
Male	Pantoprazole	Infections and infestations	127	2,566	906	17,018	0.93 (0.77-1.12)	0.453	0.562	1.000
Male	Pantoprazole	Injury, poisoning and procedural complications	134	2,559	1,189	16,735	0.74 (0.61-0.89)	0.001	0.003	0.120
Male	Pantoprazole	Investigations	294	2,399	1,847	16,077	1.07 (0.94-1.22)	0.331	0.453	1.000
Male	Pantoprazole	Metabolism and nutrition disorders	171	2,522	1,373	16,551	0.82 (0.69-0.96)	0.016	0.032	1.000
Male	Pantoprazole	Musculoskeletal and connective tissue disorders	190	2,503	1,210	16,714	1.05 (0.89-1.23)	0.558	0.648	1.000
Male	Pantoprazole	Neoplasms benign, malignant and unspecified (incl cysts and polyps)	59	2,634	625	17,299	0.62 (0.47-0.81)	<0.001	0.002	0.056
Male	Pantoprazole	Nervous system disorders	284	2,409	1,823	16,101	1.04 (0.91-1.19)	0.549	0.645	1.000
Male	Pantoprazole	Pregnancy, puerperium and perinatal conditions	3	2,690	30	17,894	0.67 (0.20-2.18)	0.501	0.601	1.000
Male	Pantoprazole	Product issues	27	2,666	131	17,793	1.38 (0.91-2.09)	0.133	0.200	1.000
Male	Pantoprazole	Psychiatric disorders	146	2,547	1,151	16,773	0.84 (0.70-1.00)	0.047	0.079	1.000
Male	Pantoprazole	Renal and urinary disorders	795	1,898	7,723	10,201	0.55 (0.51-0.60)	<0.001	<0.001	<0.001
Male	Pantoprazole	Reproductive system and breast disorders	36	2,657	207	17,717	1.16 (0.81-1.66)	0.415	0.533	1.000
Male	Pantoprazole	Respiratory, thoracic and mediastinal disorders	241	2,452	1,235	16,689	1.33 (1.15-1.53)	<0.001	<0.001	0.013
Male	Pantoprazole	Skin and subcutaneous tissue disorders	381	2,312	2,210	15,714	1.17 (1.04-1.32)	0.008	0.017	0.866
Male	Pantoprazole	Social circumstances	8	2,685	116	17,808	0.46 (0.22-0.94)	0.033	0.056	1.000
Male	Pantoprazole	Surgical and medical procedures	24	2,669	438	17,486	0.36 (0.24-0.54)	<0.001	<0.001	<0.001
Male	Pantoprazole	Vascular disorders	120	2,573	787	17,137	1.02 (0.83-1.24)	0.878	0.948	1.000
Male	Omeprazole	Blood and lymphatic system disorders	337	2,356	2,533	21,896	1.24 (1.10-1.40)	<0.001	0.002	0.065
Male	Omeprazole	Cardiac disorders	133	2,560	1,493	22,936	0.80 (0.67-0.96)	0.015	0.031	1.000
Male	Omeprazole	Congenital, familial and genetic disorders	10	2,683	94	24,335	0.96 (0.50-1.85)	0.915	0.959	1.000
Male	Omeprazole	Ear and labyrinth disorders	22	2,671	320	24,109	0.62 (0.40-0.96)	0.031	0.055	1.000
Male	Omeprazole	Endocrine disorders	55	2,638	764	23,665	0.65 (0.49-0.85)	0.002	0.005	0.210

Stratum	Comparator	System Organ Class	a	b	c	d	ROR (95% CI)	p	q (BH-FDR)	p (Bonferroni)
Male	Omeprazole	Eye disorders	84	2,609	547	23,882	1.41 (1.11-1.77)	0.004	0.009	0.455
Male	Omeprazole	Gastrointestinal disorders	515	2,178	5,392	19,037	0.83 (0.75-0.92)	<0.001	0.001	0.048
Male	Omeprazole	General disorders and administration site conditions	445	2,248	4,479	19,950	0.88 (0.79-0.98)	0.021	0.039	1.000
Male	Omeprazole	Hepatobiliary disorders	232	2,461	1,017	23,412	2.17 (1.87-2.52)	<0.001	<0.001	<0.001
Male	Omeprazole	Immune system disorders	45	2,648	576	23,853	0.70 (0.52-0.96)	0.024	0.045	1.000
Male	Omeprazole	Infections and infestations	127	2,566	1,289	23,140	0.89 (0.74-1.07)	0.215	0.309	1.000
Male	Omeprazole	Injury, poisoning and procedural complications	134	2,559	2,067	22,362	0.57 (0.47-0.68)	<0.001	<0.001	<0.001
Male	Omeprazole	Investigations	294	2,399	2,423	22,006	1.11 (0.98-1.27)	0.102	0.161	1.000
Male	Omeprazole	Metabolism and nutrition disorders	171	2,522	2,100	22,329	0.72 (0.61-0.85)	<0.001	<0.001	0.008
Male	Omeprazole	Musculoskeletal and connective tissue disorders	190	2,503	1,841	22,588	0.93 (0.80-1.09)	0.368	0.491	1.000
Male	Omeprazole	Neoplasms benign, malignant and unspecified (incl cysts and polyps)	59	2,634	1,044	23,385	0.50 (0.38-0.65)	<0.001	<0.001	<0.001
Male	Omeprazole	Nervous system disorders	284	2,409	2,567	21,862	1.00 (0.88-1.14)	0.952	0.968	1.000
Male	Omeprazole	Pregnancy, puerperium and perinatal conditions	3	2,690	39	24,390	0.70 (0.22-2.26)	0.548	0.645	1.000
Male	Omeprazole	Product issues	27	2,666	292	24,137	0.84 (0.56-1.24)	0.379	0.500	1.000
Male	Omeprazole	Psychiatric disorders	146	2,547	1,725	22,704	0.75 (0.63-0.90)	0.001	0.004	0.161
Male	Omeprazole	Renal and urinary disorders	795	1,898	11,513	12,916	0.47 (0.43-0.51)	<0.001	<0.001	<0.001
Male	Omeprazole	Reproductive system and breast disorders	36	2,657	376	24,053	0.87 (0.61-1.22)	0.416	0.533	1.000
Male	Omeprazole	Respiratory, thoracic and mediastinal disorders	241	2,452	1,642	22,787	1.36 (1.18-1.57)	<0.001	<0.001	0.002
Male	Omeprazole	Skin and subcutaneous tissue disorders	381	2,312	2,411	22,018	1.50 (1.34-1.69)	<0.001	<0.001	<0.001
Male	Omeprazole	Social circumstances	8	2,685	191	24,238	0.38 (0.19-0.77)	0.007	0.015	0.770
Male	Omeprazole	Surgical and medical procedures	24	2,669	541	23,888	0.40 (0.26-0.60)	<0.001	<0.001	0.001
Male	Omeprazole	Vascular disorders	120	2,573	1,100	23,329	0.99 (0.82-1.20)	0.911	0.959	1.000
Male	Lansoprazole	Blood and lymphatic system disorders	337	2,356	1,884	13,861	1.05 (0.93-1.19)	0.419	0.533	1.000
Male	Lansoprazole	Cardiac disorders	133	2,560	846	14,899	0.91 (0.76-1.10)	0.353	0.477	1.000
Male	Lansoprazole	Congenital, familial and genetic disorders	10	2,683	64	15,681	0.91 (0.47-1.78)	0.790	0.870	1.000
Male	Lansoprazole	Ear and labyrinth disorders	22	2,671	136	15,609	0.95 (0.60-1.49)	0.808	0.881	1.000
Male	Lansoprazole	Endocrine disorders	55	2,638	435	15,310	0.73 (0.55-0.97)	0.032	0.056	1.000
Male	Lansoprazole	Eye disorders	84	2,609	264	15,481	1.89 (1.47-2.42)	<0.001	<0.001	<0.001
Male	Lansoprazole	Gastrointestinal disorders	515	2,178	3,491	12,254	0.83 (0.75-0.92)	<0.001	0.001	0.043
Male	Lansoprazole	General disorders and administration site conditions	445	2,248	2,149	13,596	1.25 (1.12-1.40)	<0.001	<0.001	0.008
Male	Lansoprazole	Hepatobiliary disorders	232	2,461	636	15,109	2.24 (1.92-2.62)	<0.001	<0.001	<0.001
Male	Lansoprazole	Immune system disorders	45	2,648	334	15,411	0.78 (0.57-1.07)	0.129	0.197	1.000
Male	Lansoprazole	Infections and infestations	127	2,566	865	14,880	0.85 (0.70-1.03)	0.099	0.159	1.000
Male	Lansoprazole	Injury, poisoning and procedural complications	134	2,559	898	14,847	0.87 (0.72-1.04)	0.129	0.197	1.000
Male	Lansoprazole	Investigations	294	2,399	1,182	14,563	1.51 (1.32-1.73)	<0.001	<0.001	<0.001
Male	Lansoprazole	Metabolism and nutrition disorders	171	2,522	993	14,752	1.01 (0.85-1.19)	0.932	0.959	1.000
Male	Lansoprazole	Musculoskeletal and connective tissue disorders	190	2,503	828	14,917	1.37 (1.16-1.61)	<0.001	<0.001	0.018
Male	Lansoprazole	Neoplasms benign, malignant and unspecified (incl cysts and polyps)	59	2,634	620	15,125	0.55 (0.42-0.72)	<0.001	<0.001	0.001
Male	Lansoprazole	Nervous system disorders	284	2,409	1,070	14,675	1.62 (1.41-1.86)	<0.001	<0.001	<0.001
Male	Lansoprazole	Pregnancy, puerperium and perinatal conditions	3	2,690	14	15,731	1.25 (0.36-4.36)	0.723	0.813	1.000
Male	Lansoprazole	Product issues	27	2,666	69	15,676	2.30 (1.47-3.60)	<0.001	<0.001	0.028
Male	Lansoprazole	Psychiatric disorders	146	2,547	781	14,964	1.10 (0.92-1.32)	0.312	0.437	1.000

Stratum	Comparator	System Organ Class	a	b	c	d	ROR (95% CI)	p	q (BH-FDR)	p (Bonferroni)
Male	Lansoprazole	Renal and urinary disorders	795	1,898	8,952	6,793	0.32 (0.29-0.35)	<0.001	<0.001	<0.001
Male	Lansoprazole	Reproductive system and breast disorders	36	2,657	222	15,523	0.95 (0.66-1.35)	0.765	0.852	1.000
Male	Lansoprazole	Respiratory, thoracic and mediastinal disorders	241	2,452	968	14,777	1.50 (1.29-1.74)	<0.001	<0.001	<0.001
Male	Lansoprazole	Skin and subcutaneous tissue disorders	381	2,312	1,571	14,174	1.49 (1.32-1.68)	<0.001	<0.001	<0.001
Male	Lansoprazole	Social circumstances	8	2,685	75	15,670	0.62 (0.30-1.29)	0.203	0.297	1.000
Male	Lansoprazole	Surgical and medical procedures	24	2,669	575	15,170	0.24 (0.16-0.36)	<0.001	<0.001	<0.001
Male	Lansoprazole	Vascular disorders	120	2,573	589	15,156	1.20 (0.98-1.47)	0.075	0.124	1.000

a, rabeprazole ICSRs reporting the SOC; b, other rabeprazole ICSRs; c, comparator ICSRs reporting the SOC; d, other comparator ICSRs. Denominators (a + b, c + d) are substance-level numbers of individual cases, never SOC column totals, because one ICSR can report reactions in several SOCs. p, two-sided Wald test of ln(ROR); q, Benjamini-Hochberg false discovery rate-adjusted p-value over the 108 tests of the analysis family; p (Bonferroni), p × 108 (capped at 1); exact p-values are given in the accompanying Excel file. BH-FDR, Benjamini-Hochberg false discovery rate; CI, confidence interval; ICSR, individual case safety report; ROR, reporting odds ratio; SOC, MedDRA System Organ Class.

Table S5b. Female/male ratio of reporting odds ratios (test of interaction between two independent estimates; Benjamini-Hochberg adjustment over the 108 tests).

Comparator	System Organ Class	Female ROR	Male ROR	Ratio of RORs, female/male (95% CI)	p	q (BH-FDR)
Esomeprazole	Blood and lymphatic system disorders	1.06	1.11	0.96 (0.81-1.13)	0.607	0.796
Esomeprazole	Cardiac disorders	0.53	0.74	0.71 (0.55-0.93)	0.012	0.050
Esomeprazole	Congenital, familial and genetic disorders	0.26	1.04	0.25 (0.08-0.84)	0.024	0.082
Esomeprazole	Ear and labyrinth disorders	0.80	0.51	1.58 (0.93-2.68)	0.093	0.229
Esomeprazole	Endocrine disorders	0.49	0.56	0.86 (0.59-1.25)	0.430	0.636
Esomeprazole	Eye disorders	0.61	1.42	0.43 (0.31-0.60)	<0.001	<0.001
Esomeprazole	Gastrointestinal disorders	0.72	0.77	0.93 (0.82-1.06)	0.287	0.478
Esomeprazole	General disorders and administration site conditions	0.82	0.91	0.90 (0.78-1.03)	0.134	0.268
Esomeprazole	Hepatobiliary disorders	2.06	2.18	0.95 (0.77-1.17)	0.612	0.796
Esomeprazole	Immune system disorders	1.42	1.01	1.41 (0.99-2.02)	0.058	0.149
Esomeprazole	Infections and infestations	0.65	0.89	0.73 (0.57-0.93)	0.012	0.050
Esomeprazole	Injury, poisoning and procedural complications	0.40	0.39	1.03 (0.82-1.28)	0.816	0.899
Esomeprazole	Investigations	0.89	1.32	0.68 (0.57-0.81)	<0.001	<0.001
Esomeprazole	Metabolism and nutrition disorders	0.75	0.80	0.93 (0.75-1.16)	0.532	0.728
Esomeprazole	Musculoskeletal and connective tissue disorders	0.60	0.79	0.76 (0.62-0.92)	0.007	0.035
Esomeprazole	Neoplasms benign, malignant and unspecified (incl cysts and polyps)	0.30	0.37	0.82 (0.57-1.19)	0.294	0.482
Esomeprazole	Nervous system disorders	0.87	1.05	0.83 (0.70-0.98)	0.030	0.095
Esomeprazole	Pregnancy, puerperium and perinatal conditions	1.39	0.79	1.76 (0.49-6.29)	0.385	0.586
Esomeprazole	Product issues	0.89	0.98	0.90 (0.53-1.53)	0.704	0.840
Esomeprazole	Psychiatric disorders	0.63	0.76	0.84 (0.67-1.05)	0.120	0.255
Esomeprazole	Renal and urinary disorders	0.47	0.42	1.12 (1.00-1.26)	0.054	0.142
Esomeprazole	Reproductive system and breast disorders	0.83	0.99	0.84 (0.51-1.37)	0.482	0.676
Esomeprazole	Respiratory, thoracic and mediastinal disorders	0.88	1.32	0.67 (0.55-0.81)	<0.001	<0.001
Esomeprazole	Skin and subcutaneous tissue disorders	2.09	1.63	1.28 (1.10-1.48)	0.001	0.010
Esomeprazole	Social circumstances	0.37	0.32	1.13 (0.46-2.77)	0.797	0.888
Esomeprazole	Surgical and medical procedures	0.50	0.41	1.22 (0.69-2.13)	0.495	0.685
Esomeprazole	Vascular disorders	0.63	0.91	0.70 (0.53-0.91)	0.008	0.042
Pantoprazole	Blood and lymphatic system disorders	0.96	1.04	0.92 (0.78-1.10)	0.372	0.574
Pantoprazole	Cardiac disorders	0.53	0.80	0.67 (0.51-0.87)	0.003	0.018
Pantoprazole	Congenital, familial and genetic disorders	0.15	0.79	0.19 (0.06-0.64)	0.007	0.035
Pantoprazole	Ear and labyrinth disorders	0.88	0.84	1.05 (0.61-1.80)	0.867	0.928
Pantoprazole	Endocrine disorders	0.71	0.81	0.87 (0.59-1.28)	0.476	0.676
Pantoprazole	Eye disorders	0.57	1.34	0.42 (0.30-0.59)	<0.001	<0.001
Pantoprazole	Gastrointestinal disorders	0.73	0.84	0.87 (0.76-0.99)	0.040	0.118
Pantoprazole	General disorders and administration site conditions	0.72	0.89	0.81 (0.70-0.93)	0.003	0.018
Pantoprazole	Hepatobiliary disorders	1.22	1.92	0.64 (0.52-0.78)	<0.001	<0.001
Pantoprazole	Immune system disorders	0.69	0.69	1.01 (0.71-1.43)	0.976	0.994
Pantoprazole	Infections and infestations	0.44	0.93	0.48 (0.37-0.61)	<0.001	<0.001
Pantoprazole	Injury, poisoning and procedural complications	0.54	0.74	0.73 (0.58-0.91)	0.006	0.035
Pantoprazole	Investigations	0.58	1.07	0.54 (0.45-0.65)	<0.001	<0.001
Pantoprazole	Metabolism and nutrition disorders	0.66	0.82	0.81 (0.65-1.00)	0.051	0.138
Pantoprazole	Musculoskeletal and connective tissue disorders	0.56	1.05	0.53 (0.43-0.66)	<0.001	<0.001

Comparator	System Organ Class	Female ROR	Male ROR	Ratio of RORs, female/male (95% CI)	p	q (BH-FDR)
Pantoprazole	Neoplasms benign, malignant and unspecified (incl cysts and polyps)	0.42	0.62	0.68 (0.47-0.99)	0.045	0.128
Pantoprazole	Nervous system disorders	0.77	1.04	0.74 (0.63-0.88)	<0.001	0.005
Pantoprazole	Pregnancy, puerperium and perinatal conditions	0.29	0.67	0.44 (0.12-1.56)	0.202	0.376
Pantoprazole	Product issues	0.74	1.38	0.54 (0.31-0.92)	0.023	0.081
Pantoprazole	Psychiatric disorders	0.55	0.84	0.66 (0.52-0.82)	<0.001	0.003
Pantoprazole	Renal and urinary disorders	0.61	0.55	1.09 (0.97-1.23)	0.130	0.265
Pantoprazole	Reproductive system and breast disorders	1.26	1.16	1.09 (0.65-1.81)	0.754	0.866
Pantoprazole	Respiratory, thoracic and mediastinal disorders	0.79	1.33	0.60 (0.49-0.73)	<0.001	<0.001
Pantoprazole	Skin and subcutaneous tissue disorders	1.21	1.17	1.03 (0.89-1.20)	0.681	0.840
Pantoprazole	Social circumstances	0.27	0.46	0.59 (0.24-1.46)	0.255	0.438
Pantoprazole	Surgical and medical procedures	0.33	0.36	0.91 (0.52-1.59)	0.734	0.852
Pantoprazole	Vascular disorders	0.51	1.02	0.50 (0.38-0.66)	<0.001	<0.001
Omeprazole	Blood and lymphatic system disorders	1.08	1.24	0.87 (0.74-1.04)	0.120	0.255
Omeprazole	Cardiac disorders	0.64	0.80	0.80 (0.62-1.04)	0.103	0.236
Omeprazole	Congenital, familial and genetic disorders	0.25	0.96	0.26 (0.08-0.85)	0.026	0.084
Omeprazole	Ear and labyrinth disorders	0.90	0.62	1.45 (0.85-2.46)	0.173	0.334
Omeprazole	Endocrine disorders	0.56	0.65	0.87 (0.60-1.27)	0.466	0.670
Omeprazole	Eye disorders	0.73	1.41	0.52 (0.37-0.71)	<0.001	<0.001
Omeprazole	Gastrointestinal disorders	0.83	0.83	0.99 (0.87-1.13)	0.887	0.930
Omeprazole	General disorders and administration site conditions	0.90	0.88	1.02 (0.89-1.17)	0.773	0.873
Omeprazole	Hepatobiliary disorders	2.12	2.17	0.98 (0.79-1.21)	0.841	0.917
Omeprazole	Immune system disorders	1.43	0.70	2.04 (1.43-2.90)	<0.001	<0.001
Omeprazole	Infections and infestations	0.74	0.89	0.83 (0.65-1.06)	0.138	0.272
Omeprazole	Injury, poisoning and procedural complications	0.64	0.57	1.12 (0.90-1.40)	0.299	0.482
Omeprazole	Investigations	0.90	1.11	0.81 (0.68-0.97)	0.020	0.078
Omeprazole	Metabolism and nutrition disorders	0.69	0.72	0.96 (0.77-1.18)	0.687	0.840
Omeprazole	Musculoskeletal and connective tissue disorders	0.73	0.93	0.79 (0.64-0.96)	0.021	0.078
Omeprazole	Neoplasms benign, malignant and unspecified (incl cysts and polyps)	0.45	0.50	0.90 (0.62-1.31)	0.588	0.784
Omeprazole	Nervous system disorders	0.93	1.00	0.92 (0.78-1.09)	0.353	0.560
Omeprazole	Pregnancy, puerperium and perinatal conditions	1.03	0.70	1.48 (0.42-5.20)	0.543	0.733
Omeprazole	Product issues	0.67	0.84	0.80 (0.47-1.35)	0.398	0.597
Omeprazole	Psychiatric disorders	0.66	0.75	0.88 (0.70-1.10)	0.256	0.438
Omeprazole	Renal and urinary disorders	0.50	0.47	1.07 (0.95-1.20)	0.250	0.438
Omeprazole	Reproductive system and breast disorders	0.96	0.87	1.10 (0.67-1.81)	0.695	0.840
Omeprazole	Respiratory, thoracic and mediastinal disorders	1.06	1.36	0.78 (0.64-0.94)	0.010	0.044
Omeprazole	Skin and subcutaneous tissue disorders	1.85	1.50	1.23 (1.06-1.42)	0.006	0.035
Omeprazole	Social circumstances	0.53	0.38	1.41 (0.57-3.47)	0.458	0.668
Omeprazole	Surgical and medical procedures	0.43	0.40	1.08 (0.62-1.90)	0.776	0.873
Omeprazole	Vascular disorders	0.71	0.99	0.72 (0.55-0.94)	0.016	0.066
Lansoprazole	Blood and lymphatic system disorders	1.02	1.05	0.97 (0.81-1.15)	0.708	0.840
Lansoprazole	Cardiac disorders	0.87	0.91	0.95 (0.72-1.24)	0.706	0.840
Lansoprazole	Congenital, familial and genetic disorders	0.23	0.91	0.25 (0.07-0.82)	0.023	0.081
Lansoprazole	Ear and labyrinth disorders	1.48	0.95	1.56 (0.89-2.73)	0.116	0.255
Lansoprazole	Endocrine disorders	0.68	0.73	0.92 (0.63-1.35)	0.678	0.840

Comparator	System Organ Class	Female ROR	Male ROR	Ratio of RORs, female/male (95% CI)	p	q (BH-FDR)
Lansoprazole	Eye disorders	1.41	1.89	0.75 (0.53-1.05)	0.097	0.229
Lansoprazole	Gastrointestinal disorders	0.83	0.83	1.00 (0.87-1.14)	0.965	0.994
Lansoprazole	General disorders and administration site conditions	1.36	1.25	1.09 (0.94-1.26)	0.265	0.447
Lansoprazole	Hepatobiliary disorders	2.02	2.24	0.90 (0.73-1.12)	0.360	0.563
Lansoprazole	Immune system disorders	1.27	0.78	1.62 (1.13-2.32)	0.009	0.042
Lansoprazole	Infections and infestations	0.80	0.85	0.94 (0.73-1.21)	0.630	0.810
Lansoprazole	Injury, poisoning and procedural complications	1.00	0.87	1.16 (0.92-1.46)	0.208	0.381
Lansoprazole	Investigations	1.23	1.51	0.82 (0.68-0.99)	0.035	0.106
Lansoprazole	Metabolism and nutrition disorders	1.02	1.01	1.02 (0.82-1.27)	0.877	0.928
Lansoprazole	Musculoskeletal and connective tissue disorders	1.19	1.37	0.87 (0.70-1.08)	0.202	0.376
Lansoprazole	Neoplasms benign, malignant and unspecified (incl cysts and polyps)	0.53	0.55	0.97 (0.67-1.41)	0.876	0.928
Lansoprazole	Nervous system disorders	1.41	1.62	0.87 (0.73-1.04)	0.128	0.265
Lansoprazole	Pregnancy, puerperium and perinatal conditions	1.26	1.25	1.01 (0.27-3.82)	0.992	0.995
Lansoprazole	Product issues	1.40	2.30	0.61 (0.34-1.09)	0.093	0.229
Lansoprazole	Psychiatric disorders	1.10	1.10	1.00 (0.79-1.27)	0.995	0.995
Lansoprazole	Renal and urinary disorders	0.32	0.32	1.02 (0.91-1.15)	0.715	0.840
Lansoprazole	Reproductive system and breast disorders	1.65	0.95	1.74 (1.03-2.93)	0.037	0.110
Lansoprazole	Respiratory, thoracic and mediastinal disorders	1.34	1.50	0.89 (0.73-1.09)	0.251	0.438
Lansoprazole	Skin and subcutaneous tissue disorders	1.73	1.49	1.17 (1.00-1.36)	0.046	0.129
Lansoprazole	Social circumstances	0.63	0.62	1.01 (0.40-2.56)	0.976	0.994
Lansoprazole	Surgical and medical procedures	0.38	0.24	1.61 (0.92-2.83)	0.096	0.229
Lansoprazole	Vascular disorders	0.96	1.20	0.80 (0.60-1.06)	0.118	0.255

Ratio of RORs = female ROR/male ROR; its 95% CI and z-test use the standard errors of the two log RORs. BH-FDR, Benjamini-Hochberg false discovery rate; ROR, reporting odds ratio.
